# The dCache Domain of the Chemoreceptor Tlp1 in Campylobacter jejuni Binds and Triggers Chemotaxis toward Formate

**DOI:** 10.1128/mbio.03564-22

**Published:** 2023-04-13

**Authors:** Jingjing Duan, Qi Zhao, Yuxin Wang, Zhe Chi, Wei Li, Xue Wang, Shuangjiang Liu, Shuangyu Bi

**Affiliations:** a State Key Laboratory of Microbial Biotechnology, Shandong University, Qingdao, China; b State Key Laboratory of Microbial Resources, and Environmental Microbiology Research Center, Institute of Microbiology, Chinese Academy of Sciences, Beijing, China; c College of Marine Life Sciences, Ocean University of China, Qingdao, China; d Department of Clinical Laboratory, Qilu Hospital, Shandong University, Jinan, China; Max-Planck-Institut fur terrestrische Mikrobiologie

**Keywords:** *Campylobacter jejuni*, chemotaxis, Tlp1, formate, hybrid receptor

## Abstract

Chemotaxis is an important virulence factor in some enteric pathogens, and it is involved in the pathogenesis and colonization of the host. However, there is limited knowledge regarding the environmental signals that promote chemotactic behavior and the sensing of these signals by chemoreceptors. To date, there is no information on the ligand molecule that directly binds to and is sensed by Campylobacter jejuni Tlp1, which is a chemoreceptor with a dCache-type ligand-binding domain (LBD). dCache (**d**ouble **Ca**lcium channels and **che**motaxis receptor) is the largest group of sensory domains in bacteria, but the dCache-type chemoreceptor that directly binds to formate has not yet been discovered. In this study, formate was identified as a direct-binding ligand of C. jejuni Tlp1 with high sensing specificity. We used the strategy of constructing a functional hybrid receptor of C. jejuni Tlp1 and the Escherichia coli chemoreceptor Tar to screen for the potential ligand of Tlp1, with the binding of formate to Tlp1-LBD being verified using isothermal titration calorimetry. Molecular docking and experimental analyses indicated that formate binds to the membrane-proximal pocket of the dCache subdomain. Chemotaxis assays demonstrated that formate elicits robust attractant responses of the C. jejuni strain NCTC 11168, specifically via Tlp1. The chemoattraction effect of formate via Tlp1 promoted the growth of C. jejuni, especially when competing with Tlp1- or CheY-knockout strains. Our study reveals the molecular mechanisms by which C. jejuni mediates chemotaxis toward formate, and, to our knowledge, is the first report on the high-specificity binding of the dCache-type chemoreceptor to formate as well as the physiological role of chemotaxis toward formate.

## INTRODUCTION

Campylobacter jejuni is the leading cause of acute bacterial gastroenteritis in both developing and industrialized countries (1). As a commensal bacterium in the gastrointestinal tracts of poultry and other birds, C. jejuni is mainly transmitted to humans through contaminated food and causes severe gastrointestinal diseases, including watery or bloody inflammatory diarrhea and complications (2, 3). The molecular mechanisms of C. jejuni pathogenesis and virulence are still poorly understood. Previous studies have demonstrated that flagellar motility and chemotaxis are important virulence factors in C. jejuni that promote adhesion to and the colonization of host epithelial cells (4, 5). However, little is known about the chemoeffectors detected by the C. jejuni chemotaxis system and their physiological functions.

Chemotaxis enables motile bacterial navigation in environmental gradients of chemical substances so that they can find optimal niches for their proliferation (6, 7). Signal molecules in the environment are sensed as attractants or repellents by a repertoire of chemoreceptors (also termed methyl-accepting chemotaxis proteins [MCPs] or transducer-like proteins [Tlps]), and they control the activity of the histidine kinase CheA. Activated CheA transfers a phosphoryl group to the response regulator CheY. Phosphorylated CheY interacts with flagellar motor(s) to change the rotational direction of flagella, thereby allowing bacteria to swim toward attractants or away from repellents (8). In addition, the scaffold proteins CheW, methyltransferase CheR, and methylesterase CheB are core chemotaxis proteins that are present in almost all chemosensory pathways (6).

The C. jejuni strain NCTC 11168 contains 10 putative chemoreceptors that can be divided into different groups, according to their topologies (9, 10). Chemoreceptors Tlp1 to Tlp4, Tlp7, and Tlp10 belong to the class I topology group, which is composed of a periplasmic ligand-binding domain (LBD), two transmembrane helices, and a cytoplasmic signaling domain (11). Chemoeffectors are commonly perceived to bind directly to LBDs as ligands or to interact with other elements of the receptors (12, 13). However, the discovery of chemoeffectors and the functional annotation of chemoreceptors remain challenging tasks. At present, the ligand specificities of only a few C. jejuni chemoreceptors have been clarified (14–17).

The chemoreceptor Tlp1 (*Cj1506c*) is the most conserved chemoreceptor among C. jejuni strains (18). The crystal structure of Tlp1-LBD indicates that it belongs to the double calcium channels and chemotaxis receptor (dCache) domain (18), which is the largest group of sensory domains in bacteria and can accommodate various types of ligands (12, 19). Both the membrane-proximal and membrane-distal subdomains of dCache contain a ligand-binding pocket (14, 20, 21). For the large majority of dCache domains, signals bind to the membrane-distal subdomain (14, 21). A few studies also showed that both subdomains can bind ligands (22, 23). The direct-binding ligand of Tlp1, which triggers chemotactic responses in C. jejuni, has not yet been identified. A previous report indicated that Tlp1 is involved in the chemoattraction response to aspartate (24); however, a subsequent study showed that aspartate does not bind to Tlp1-LBD directly (18). Acetate and chloride ions (both from the crystallization buffer) have been observed to bind to membrane-proximal and membrane-distal subdomains in the Tlp1-LBD crystal structure, respectively (18). Isothermal titration calorimetry (ITC) measurements have confirmed the weak binding of acetate (*K_d_ *= 3.4 mM) and chloride ions (*K_d_ *= 70 mM) to the Tlp1-LBD protein; however, it has been reported that acetate and chloride cannot elicit chemotaxis in C. jejuni (25). Therefore, they are unlikely to be the natural ligands of Tlp1.

Tlp1 is involved in the commensal colonization of the chicken intestine by C. jejuni and might play an important role in the infection and colonization of the human host, as well (26). Previous studies have indicated that the *tlp1* gene is strongly upregulated in C. jejuni strains that are colonized in chickens (27). It may also be involved in the ability of C. jejuni to attach to human intestinal cells in culture (28, 29). The *tlp1*-isogenic knockout strains had significantly reduced abilities to colonize avian and mammalian hosts, as demonstrated using chicken and mouse models (24, 28). However, as the natural ligand of Tlp1 is still unknown, the link between chemotaxis via Tlp1 and physiological functions has not been demonstrated until now.

To annotate the function of C. jejuni chemotaxis, it is necessary to develop novel strategies for the screening of the ligand molecules of the target chemoreceptor. Previous studies have shown that the construction of hybrid receptors in bacteria is a potentially powerful tool for elucidating the ligand specificity of the chemoreceptor of interest, with the LBD from the “donor” chemoreceptor reliably being coupled to the cytoplasmic part of the Escherichia coli (“recipient”) chemoreceptor (30). Because the designed hybrid receptor controls the chemotaxis system of E. coli, several standardized chemotaxis assays can be used to characterize the specificity of the target LBD. These approaches allow for the screening of novel ligands of chemoreceptors as well as the quantification of the chemotactic abilities of the proposed ligands.

Although the dCache domains have been reported to accommodate various types of ligands (12), the dCache-type chemoreceptor that directly binds to formate and elicits the chemotaxis of bacteria has not yet been discovered. Previously, the short-chain carboxylate-sensing chemoreceptor McpV with the sCache LBD from Sinorhizobium meliloti was reported to bind formate with low affinity (*K_d_ *= 8.7 mM). It could not trigger chemotaxis until the concentration of formate reached 100 mM, indicating that formate is not a primary ligand of McpV and is an inefficient chemoattractant for S. meliloti (31). Until now, the chemoreceptor Atu0526 with an sCache LBD from Agrobacterium fabrum C58 was the only receptor that was reported to bind formate with higher affinity (*K_d_ *= 172 μM) and trigger chemotaxis (32).

In the present study, we identified the direct-binding ligand of Tlp1 that triggers strong chemotaxis and revealed the physiological role of Tlp1-mediated chemotaxis toward the ligand on the growth of C. jejuni. We used the strategy of constructing a hybrid receptor of C. jejuni Tlp1 and the E. coli chemoreceptor Tar to screen for the ligand of Tlp1 using microfluidic assays. Formate was identified as a potential chemoeffector that was specifically sensed by the Tlp1-LBD. Molecular docking predictions and ITC measurements showed that it binds directly to the membrane-proximal pocket of Tlp1-LBD. Chemotaxis assays indicated that formate elicited a robust attractant response in C. jejuni NCTC 11168 via Tlp1. We further elucidated that the Tlp1-mediated chemoattractant effect of formate promotes the growth of C. jejuni, especially when competing with the Tlp1- or CheY-knockout strains. Our study reveals the molecular mechanisms by which C. jejuni mediates chemotaxis toward formate. To the best of our knowledge, this is the first report on the high-specificity binding of the dCache-type chemoreceptor to formate as well as the physiological function of chemotaxis toward formate.

## RESULTS

### Construction of a functional Tlp1-Tar hybrid chemoreceptor for ligand screening.

**FIG 1:**
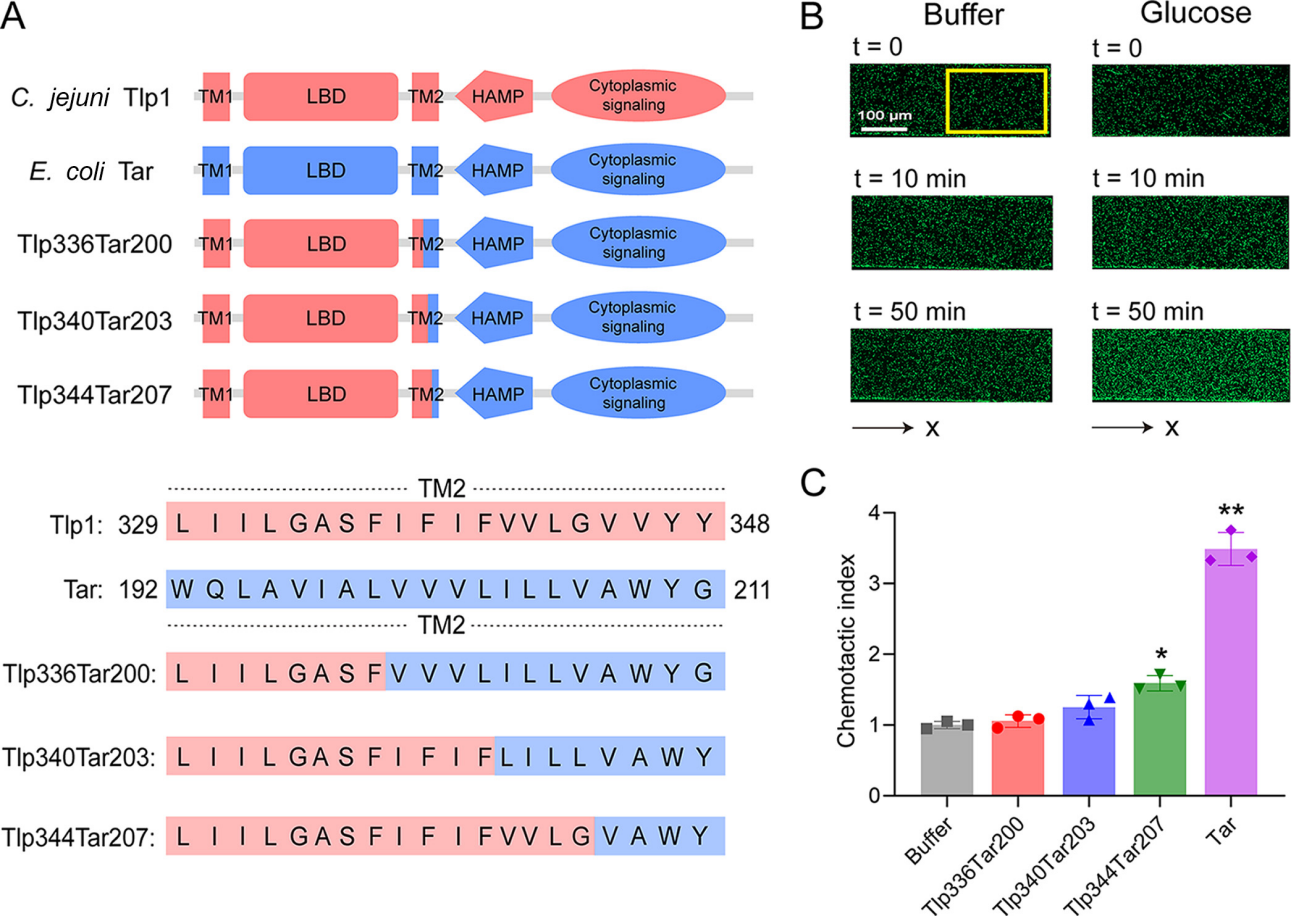
Design and construction of the functional Tlp1-Tar hybrid chemoreceptors. (A) Design and construction of the hybrid receptors Tlp336Tar200, Tlp340Tar203, and Tlp344Tar207. The upper panel shows the architecture of Tlp1 (red), Tar (blue), and the Tlp1-Tar hybrid receptor with a periplasmic LBD, two transmembrane helixes (TM1, TM2), HAMP domain, and cytoplasmic signaling domain. The lower panel shows the sequence alignment for Tlp1 and Tar, shown in red and blue, respectively, with the sequences of the hybrid receptors given below. (B) Examples of the distribution of E. coli cells expressing Tlp344Tar207 in the observation channel of the microfluidic device, acquired before the addition of ligands as well as 10 min and 50 min after the addition of 30 mM glucose at the source pore (scale bar: 100 μm). The *x* component (black arrow) indicates the direction up the concentration gradient of glucose. The response is characterized by measurements of the fluorescence intensity (cell density) in the analysis region (150 × 300 μm) of the observation channel, which is indicated by a yellow rectangle. (C) Relative fluorescence intensities of the cells expressing Tlp336Tar200, Tlp340Tar203, Tlp344Tar207, or Tar as the sole receptor in the analysis region of the observation channel at 50 min after the addition of glucose at the source or without ligand (buffer). The corresponding values of the fluorescence intensities in the analysis regions were normalized to the fluorescence intensity of the cells in the buffer to obtain the chemotactic index values. Error bars indicate the standard errors of three replicates. The *P* values were calculated using a paired *t* test. *, *P* < 0.05; **, *P* < 0.01, compared to the buffer. LBD, ligand-binding domain; HAMP, histidine kinases, adenylate cyclases, methyl-accepting proteins, and phosphatases.

To screen for potential ligands of C. jejuni Tlp1, we designed and constructed hybrid chemoreceptors with the Tlp1-LBD fused to the cytoplasmic domain of the E. coli chemoreceptor Tar. Three Tlp1-Tar hybrid receptors with different fusion positions in the second transmembrane helix (TM2) were obtained: Tlp1(1 to 336)-Tar(200 to 553) (Tlp336Tar200), Tlp1(1 to 340)-Tar(203 to 553) (Tlp340Tar203), and Tlp1(1 to 344)-Tar(207 to 553) (Tlp344Tar207) (Fig. 1A).

To characterize the activity of these hybrid receptors, we measured the responses of the E. coli receptorless strain VS188 expressing each hybrid as the sole receptor and the green fluorescent protein (GFP) to a concentration gradient of glucose, which is a substrate of the phosphotransferase system (PTS) that stimulates the E. coli functional receptor-CheA-CheW ternary complex and triggers chemoattractant responses (6, 33) (Fig. S1) using a previously reported, modified microfluidic device (30) (Fig. S2). The PTS-mediated influx of glucose into the cell lowers the phosphorylation state of the PTS proteins, which then inhibit CheA activity, apparently by interacting with the cytoplasmic side of the chemosensory complexes. Such chemotactic responses that are triggered by the PTS are independent of the chemoreceptor LBDs (34). E. coli cells were loaded into the sink pore of the device and allowed to swim into the observation channel. The compound solution was then loaded into the source pore and gradually diffused into the observation channel to form a concentration gradient. If the compound is an attractant, bacterial cells move from the sink pore and accumulate in the observation channel, thereby increasing cell intensity. If the compound is a repellent, cells move out of the observation channel toward the sink pore, thereby decreasing cell intensity.

10.1128/mbio.03564-22.1 FIG S1 PTS links chemotactic response to the uptake of sugars. Chemotactic sensing is linked to the uptake of various sugars, such as glucose, through the phosphotransferase system (PTS). PTS-mediated sugar influx into cells reduces the phosphorylation state of PTS proteins, which in turn modulates CheA activity, apparently by interacting with the cytoplasmic side of the chemosensory complexes. This results in a change in the direction of flagellar rotation and, ultimately, in the motility behavior of E. coli. Download FIG S1, TIF file, 0.7 MB. Copyright © 2023 Duan et al. 2023 Duan et al. https://creativecommons.org/licenses/by/4.0/ This content is distributed under the terms of the Creative Commons Attribution 4.0 International license.

10.1128/mbio.03564-22.2 FIG S2 The microfluidic device used in this study. Each chip contains 24 parallel units, each of which contains a sink pore and a source pore. The sink pore and source pore are connected by 5 agarose channels (150 μm long, 20 μm wide, and 5 μm high) and an observation channel (600 μm long, 200 μm wide, and 18 μm high). Download FIG S2, TIF file, 0.5 MB. Copyright © 2023 Duan et al. 2023 Duan et al. https://creativecommons.org/licenses/by/4.0/ This content is distributed under the terms of the Creative Commons Attribution 4.0 International license.

We observed that the GFP-labeled E. coli cells expressing Tlp344Tar207 exhibited the strongest chemoattractant response to the glucose gradient among the three hybrid receptors, with cells drifting up the glucose gradient and the accumulation of cells in the observation channel increasing over time (Fig. 1B and C). Such a chemotactic response to glucose was expressed in terms of the chemotactic index (CI), which is the corresponding value of the fluorescence intensity in the analysis region in response to glucose, normalized to the fluorescence intensity of the cells in the buffer. CI > 1 indicates a chemoattractant response, whereas CI < 1 indicates a chemorepellent response. Simultaneously, we used the chemoattractant response of E. coli cells expressing full-length Tar as the only receptor for glucose as a positive-control (Fig. 1C). Therefore, Tlp344Tar207 has a strong ability to form a functional ternary complex and stimulate the chemosensory pathway. The other two hybrid receptors, namely, Tlp336Tar200 and Tlp340Tar203, elicited much weaker responses to glucose via PTS, indicating that they are less effective in communicating with the E. coli chemosensory system. We used Tlp344Tar207 for subsequent ligand screening.

### Microfluidic screening for the potential ligand of Tlp1 using the Tlp344Tar207 receptor.

Next, candidate compounds that could be used for ligand screening were selected. Based on the reported crystal structure of Tlp1-LBD (PDB ID: 4WY9) (18), the potential ligand-binding pockets in the membrane-proximal and membrane-distal subdomains are relatively small, with the membrane-proximal pocket having an area of 603.3 Å^2^ and a volume of 28.8 Å^3^ and the membrane-distal pocket having an area of 1439.9 Å^2^ and volume of 48.7 Å^3^, as predicted using the PyVOL package of the PyMOL software tool (Fig. S3). Therefore, we selected 54 compounds with molecular weights of less than 155 Da from the Biolog compound arrays (PM1, PM2A, PM3B, and PM5) as well as compounds that are commonly present in the intestine for ligand screening (Table S1).

10.1128/mbio.03564-22.3 FIG S3 Prediction and analysis of the ligand-binding pockets of Tlp1-LBD. (A) The white areas indicate the ligand-binding pockets that are present in the membrane-distal and membrane-proximal subdomains. The top pocket is the membrane-distal pocket, whereas the bottom one is the membrane-proximal pocket. (B) The ligand-binding pockets are filled with yellow spheres to show the volumes of the pockets. Download FIG S3, TIF file, 1.5 MB. Copyright © 2023 Duan et al. 2023 Duan et al. https://creativecommons.org/licenses/by/4.0/ This content is distributed under the terms of the Creative Commons Attribution 4.0 International license.

10.1128/mbio.03564-22.9 TABLE S1 Compounds used for ligand screening in this study. Download Table S1, DOCX file, 0.1 MB. Copyright © 2023 Duan et al. 2023 Duan et al. https://creativecommons.org/licenses/by/4.0/ This content is distributed under the terms of the Creative Commons Attribution 4.0 International license.

**FIG 2:**
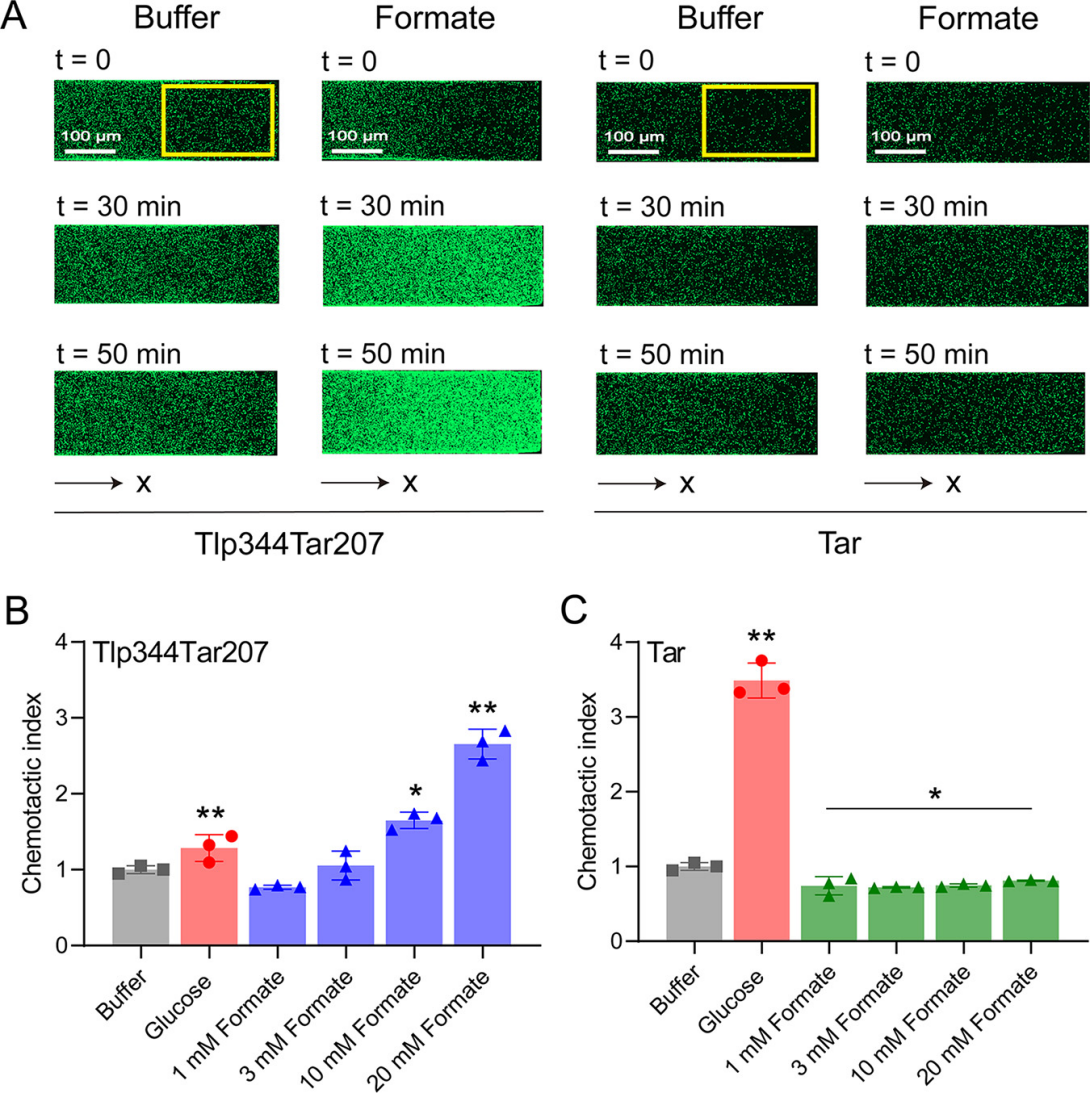
Microfluidic screening for potential ligands of Tlp1 using the Tlp344Tar207 receptor. (A) Examples of the distribution of E. coli cells expressing Tlp344Tar207 or Tar as the sole receptor in the observation channel of the microfluidic device, acquired before the addition of ligands as well as 30 min and 50 min after the addition of 20 mM formate at the source pore (scale bar: 100 μm). The *x* component (black arrow) indicates the direction up the concentration gradient of formate. The response is characterized by measurements of the fluorescence intensity (cell density) in the analysis region (150 × 300 μm) of the observation channel, which is indicated by a yellow rectangle. (B) Relative fluorescence intensity of E. coli cells expressing Tlp344Tar207 as the sole receptor in the analysis region of the observation channel at 50 min after the addition of the indicated formate concentrations at the source or without ligand (buffer). (C) Relative fluorescence intensity of E. coli cells expressing Tar as the sole receptor in the analysis region of the observation channel 50 min after the addition of the indicated formate concentrations at the source or without ligand (buffer). In panels B and C, the corresponding values of the fluorescence intensities in the analysis regions were normalized to the fluorescence intensity of the cells in the buffer to obtain the chemotactic index. Error bars indicate the standard errors of three replicates. The *P* values were calculated using a paired *t* test. *, *P* < 0.05; **, *P* < 0.01, compared to the buffer.

The responses of E. coli cells expressing Tlp344Tar207 as the sole receptor and GFP were measured to screen for potential ligands of Tlp1 from this compound library, using the microfluidic device described above. Among the compounds listed in Table S1, Tlp344Tar207 mediated a robust attractant response toward formate, with cells moving up the formate gradient and accumulating in the observation channel (Fig. 2A). This attractant response was concentration-dependent (Fig. 2B), indicating that formate was an attractant for Tlp344Tar207. To investigate whether the response toward formate was mediated by Tlp1-LBD, we measured the responses of E. coli cells expressing full-length Tar as the sole receptor to serve as a control. In contrast to Tlp344Tar207, the cells expressing the Tar receptor could not be attracted by formate but instead had slightly decreased cell densities at the tested concentrations, compared to that observed in the buffer (Fig. 2C), suggesting that formate works through the Tlp1-LBD of Tlp344Tar207 to trigger chemoattraction responses and is a Tlp1 specific chemoeffector.

### Binding interactions of formate with Tlp1-LBD.

**FIG 3:**
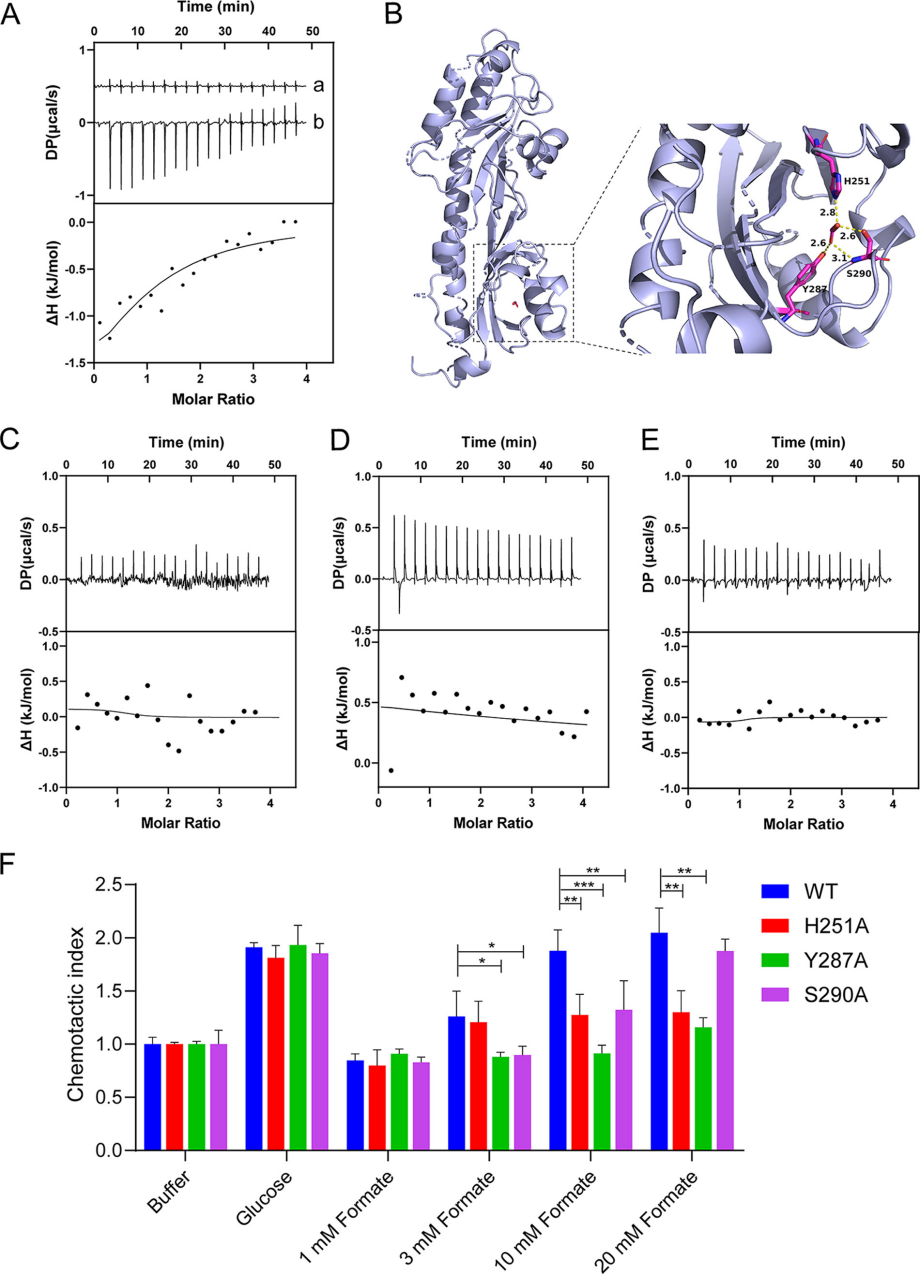
The binding of formate to Tlp1-LBD and its mutant proteins as well as the chemotaxis of Tlp344Tar207 mutants to glucose and formate. (A) Microcalorimetric titrations of Tlp1-LBD with formate at pH 8.0. “a” indicates the titration of formate to buffer, and “b” indicates that of formate to Tlp1-LBD. Upper panel, titration raw data; lower panel, fit of dilution heat-corrected and concentration-normalized raw data with a model for the binding of a single ligand to a macromolecule. The concentrations of Tlp1-LBD and formate were 210 μM and 4 mM, respectively. The curve corresponds to the best fit that was calculated using the “one binding site model” of the Malvern MicroCal PEAQ ITC Analysis software package. (B) Molecular docking analysis of Tlp1-LBD to formate, carried out using Autodock. The conformation with the lowest docking energy was rendered using PyMOL software. Formate binds to the membrane-proximal pocket of Tlp1-LBD. The key residues in the ligand-binding pocket that is involved in formate binding are shown as sticks. The hydrogen bonds are shown as yellow dashed lines. The distances between formate and residue H251, Y287, or S290 are indicated. (C–E) ITC titrations of Tlp1-LBD H251A, Y287A, and S290A with formate. The concentrations of Tlp1-LBD H251A, Y287A, and S290A were 211, 192, and 211 μM, respectively, while the concentration of formate was 4 mM. (F) Chemotaxis of Tlp344Tar207 mutants to glucose and different concentrations of formate. The relative fluorescence intensity of E. coli cells expressing Tlp344Tar207 wild-type (WT), Tlp344Tar207-H251A, -Y287A, and -S290A, respectively, at 50 min after the addition of 30 mM glucose and the indicated concentrations of formate at the source or without ligand (buffer). The corresponding values of the fluorescence intensities were normalized to the fluorescence intensity of the cells in the buffer to obtain the chemotactic index. Error bars indicate the standard errors of three replicates. *, *P* < 0.05; **, *P* < 0.01; ***, *P* < 0.001.

We measured the *in vitro* binding affinities of formate toward purified Tlp1-LBD protein (residues 31 to 326) using ITC. The titration of Tlp1-LBD with formate produced obvious thermal changes, which diminished gradually as the binding reached saturation, thereby suggesting that the binding process was driven by a favorable enthalpy change (Fig. 3A). The ITC-derived *K_d_* of Tlp1-LBD binding to formate was 245 ± 74 μM at pH 8.0, indicating that formate is a direct-binding ligand of Tlp1. We also measured the binding affinity of formate with Tlp1-LBD at pH 5.0, and the ITC-derived *K_d_* was 795 ± 86 μM (Fig. S4A), which is consistent with a previous report which showed that periplasmic LBDs recognize ligands over a broad pH range (35).

10.1128/mbio.03564-22.4 FIG S4 Microcalorimetric titrations of Tlp1-LBD with formate at pH 5.0 and measurements of circular dichroism spectroscopy for Tlp1-LBD and its mutant proteins. (A) Microcalorimetric titrations of Tlp1-LBD with formate at pH 5.0. “a” indicates the titration of formate to buffer, and “b” indicates that of formate to Tlp1-LBD. Upper panel, titration raw data; lower panel, fit of dilution heat-corrected and concentration-normalized raw data with a model for the binding of a single ligand to a macromolecule. The concentrations of Tlp1-LBD and formate were 158 μM and 3 mM, respectively. The curve corresponds to the best fit that was calculated using the “one binding site model” of the Malvern MicroCal PEAQ ITC Analysis software package. (B) The results of circular dichroism spectroscopy for Tlp1-LBD wild type, H251A, Y287A, and S290A. S290A is connected to the SUMO fusion protein. The spectrum was recorded at 25°C in desalting buffer (25 mM Na_2_HPO_4_, 25 mM NaH_2_PO_4_, pH 8.0). Download FIG S4, TIF file, 0.5 MB. Copyright © 2023 Duan et al. 2023 Duan et al. https://creativecommons.org/licenses/by/4.0/ This content is distributed under the terms of the Creative Commons Attribution 4.0 International license.

To understand how formate binds to Tlp1-LBD, molecular docking was performed to calculate the free energies of the predicted binding modes. The lowest binding free energies for the conformation of formate binding to the membrane-distal and membrane-proximal pockets were found to be −1.81 and –2.23 kcal/mol, respectively, which suggested that the membrane-proximal pocket might be the binding site for formate. A receptor-ligand interaction analysis indicated that formate makes close contact with the residues H251, Y287, and S290 in the membrane-proximal pocket, with the formation of hydrogen bonds being observed between the formate carboxyl group and the side chains of these three residues (Fig. 3B). To further confirm the contributions of H251, Y287, and S290 to formate binding, we substituted these residues with alanine, and the Tlp1-LBD mutant proteins H251A, Y287A, and S290A were generated and purified, after which the binding affinities of formate toward these proteins were analyzed using ITC. The ITC results showed that the binding affinities of formate to H251A, Y287A, and S290A were much lower than those to wild-type Tlp1-LBD (Fig. 3C–E). The measurements of circular dichroism spectroscopy indicated that the single mutation did not alter the secondary structure of H251A, Y287A, and S290A mutant proteins (Fig. S4B), suggesting that H251, Y287, and S290 are key residues for formate binding.

We also investigated whether the single mutation of H251A, Y287A, or S290A in Tlp1 could affect the chemotactic response to formate. We constructed each mutation in the Tlp344Tar207 hybrid receptor and tested its response to formate. The results showed that E. coli cells expressing each of the mutant Tlp344Tar207-H251A, -Y287A, and -S290A had a much weaker chemotactic response to formate, compared to the wild-type Tlp344Tar207, at the tested concentrations (Fig. 3F). However, their responses to glucose were similar, indicating that the single mutation did not influence the activity of the hybrid receptor. These results also suggested that the residues H251, Y287, and S290 in Tlp1 are crucial for formate sensing.

### The chemoattractant response of C. jejuni toward formate is mediated by Tlp1.

**FIG 4:**
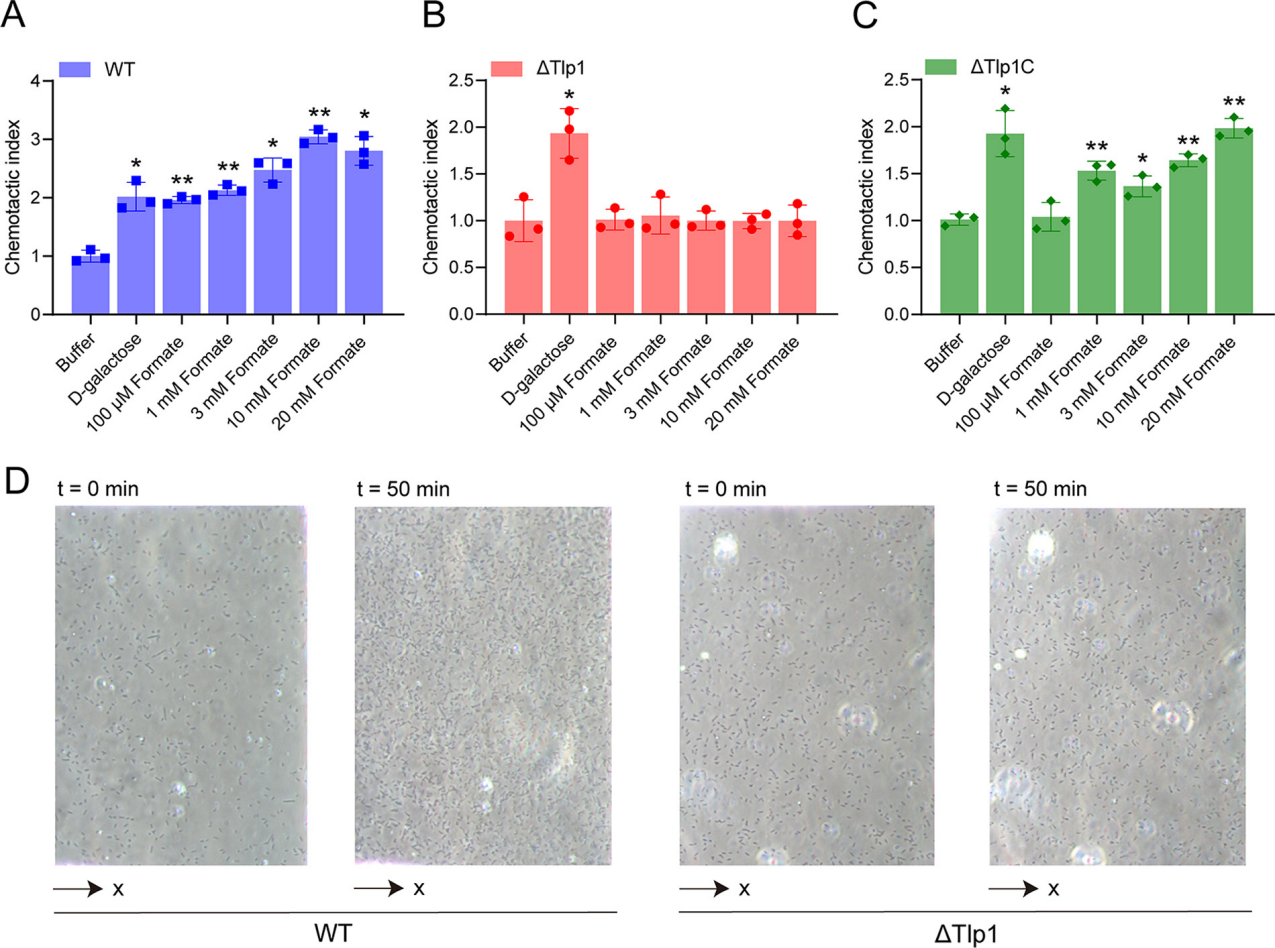
Chemotactic responses of the C. jejuni strain NCTC 11168 toward formate, as measured using microfluidics. (A–C) The responses of C. jejuni WT (A), Tlp1 knockout strain ΔTlp1 (B), and Tlp1 complement strain ΔTlp1C (C) to different concentrations of formate. The data are shown as the relative chemotactic strength (chemotactic index) in the analysis region of the observation channel at 50 min after the addition of the indicated ligand concentrations at the source or without ligand (buffer). The chemotactic index was obtained by normalizing the corresponding cell numbers in the analysis regions to the number of cells in the buffer. The response to 20 mM d-galactose was considered to be the positive control. Error bars indicate the standard errors of three replicates. Significant differences, compared to the buffer, were calculated using a paired *t* test. *, *P* < 0.05; **, *P* < 0.01. (D) Examples of WT or ΔTlp1 cell distribution in the observation channel of the microfluidic device, acquired before the addition of ligands and at 50 min after the addition of 20 mM formate at the source pore. The *x* component (black arrow) indicates the direction up the concentration gradient of formate. The response is characterized by measurements of the cell numbers in the analysis region (150 × 100 μm) of the observation channel, which is the view in panel D. WT, wild type.

Next, we measured the responses of C. jejuni strain NCTC 11168 toward formate using the microfluidic device described above. As a control to measure the chemotactic response, we used d-galactose, which is a reported attractant for C. jejuni (16). The C. jejuni wild-type (WT) cells drifted up the d-galactose gradient in the observation channel, and the number of accumulated cells in the observation channel increased over time, indicating an attractant response toward d-galactose (Fig. 4A). Similarly, C. jejuni cells exhibited a robust attractant response toward formate, with cells moving up the formate gradient, and an increase in the number of cells in the observation channel (Fig. 4A). This attractant response was concentration-dependent, indicating that formate is an attractant for C. jejuni, which is consistent with the results of previous reports (29, 36, 37).

To investigate whether the attractant response of C. jejuni toward formate is mediated by Tlp1, we measured the responses of the C. jejuni Tlp1 knockout strain (ΔTlp1) and the Tlp1 complement strain (ΔTlp1C) toward formate. In the motility phenotype measurements, ΔTlp1 showed slightly higher motility than did the WT (Fig. S5A). Meanwhile, it also showed a significant attractant response to the control, namely, d-galactose, but it failed to exhibit a chemotactic response toward formate at any of the tested concentrations (Fig. 4B). In contrast, the expression of Tlp1 (ΔTlp1C) in the corresponding mutant strain restored the attractant response toward formate (Fig. 4C), suggesting that formate works through Tlp1. As a control, the nonchemotactic mutant ΔCheY did not undergo chemotaxis toward formate or d-galactose (Fig. S5B), suggesting that the chemotactic system mediates the response toward formate. These results indicated that the attractant response of C. jejuni toward formate is dependent on the chemoreceptor Tlp1 through the chemotaxis system. In addition, we measured the responses of C. jejuni to short-chain fatty acids besides formate, including acetate and propionate, but C. jejuni did not undergo chemotaxis toward them (Fig. S5C), which is consistent with the results of both a previous report (18) and our microfluidics assay using the Tlp344Tar207 hybrid receptor.

10.1128/mbio.03564-22.5 FIG S5 Chemotaxis and motility assays of C. jejuni WT and mutant cells. (A) Motility assays for C. jejuni WT and ΔTlp1. Both the WT and ΔTlp1 strains were inoculated into 0.4% MH soft agar by pipetting the same amount of bacteria. The diameter of the swimming ring was measured. An asterisk indicates a significant difference (*P* value of <0.05) from the buffer. (B) The chemotactic index of the ΔCheY strain in response to 20 mM d-galactose and 1 mM formate, respectively. (C) The chemotactic index of the C. jejuni wild-type strain in response to 2 mM acetate and 2 mM propionate, respectively. In panels A to C, the error bars represent the standard errors of three replicates. Download FIG S5, TIF file, 0.4 MB. Copyright © 2023 Duan et al. 2023 Duan et al. https://creativecommons.org/licenses/by/4.0/ This content is distributed under the terms of the Creative Commons Attribution 4.0 International license.

Tlp1 was previously identified to respond to aspartate, and that response is most likely mediated by a periplasmic binding protein (18). We measured the chemotactic response of C. jejuni to aspartate, and the results showed that C. jejuni indeed performed an obvious attractant response to aspartate (Fig. S6). In order to explore the effect of aspartate on formate chemotaxis, we conducted competitive experiments using microfluidics. When C. jejuni cells were adapted in 10 mM aspartate, their chemotaxis to formate reduced significantly (Fig. S6), indicating that the chemotaxis to aspartate and formate might be competitive.

10.1128/mbio.03564-22.6 FIG S6 The effect of aspartate on the chemotaxis to formate. The gray columns show the chemotaxis of C. jejuni WT to 10 mM formate and 10 mM aspartate (Asp), respectively. The red columns show the results of competitive experiments. Wild-type cells were adapted in 10 mM aspartate, and then their chemotaxis to formate was measured. Significant differences, compared to the buffer, were calculated using a paired *t*-test. *, *P* < 0.05; **, *P* < 0.01; ***, *P* < 0.001. The error bars represent the standard errors of four replicates. Download FIG S6, TIF file, 0.4 MB. Copyright © 2023 Duan et al. 2023 Duan et al. https://creativecommons.org/licenses/by/4.0/ This content is distributed under the terms of the Creative Commons Attribution 4.0 International license.

### The Tlp1-mediated chemoattractant effect of formate promotes the growth of C. jejuni.

Formate is a primary energy source for C. jejuni (29, 38). It can serve as the electron donor and can be metabolized by a formate dehydrogenase (39). Electrons generated from the oxidation of formate are passed down a branching electron transport chain. A previous study showed that formate reduces oxidase activity under microaerobic conditions as well as aerotolerance under ambient oxygen conditions, whereas it increases the expression of genes encoding the proteins that facilitate the use of alternative electron acceptors (38). Formate possibly facilitates the shuttling of electrons to alternative acceptors while likely conserving limited oxygen concentrations for other essential functions so that it plays a role in optimizing the adaptation of C. jejuni to the microaerobic conditions.

**FIG 5:**
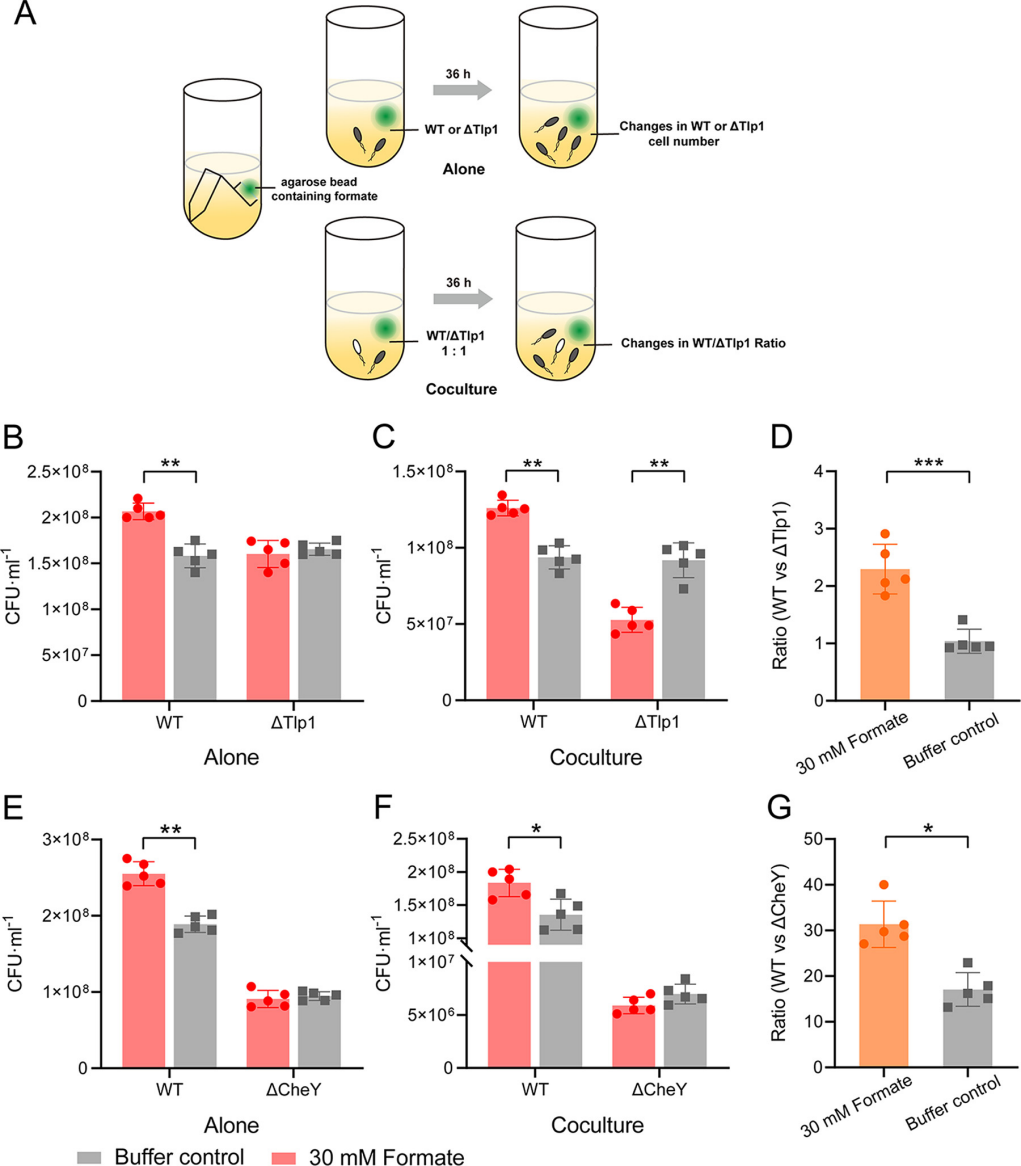
Role of Tlp1-mediated chemotaxis toward formate in the growth of C. jejuni. (A) Schematic representation of the experimental design used to generate the formate gradient in the unstirred culture. (B) Bacterial numbers of C. jejuni WT and ΔTlp1 cells grown individually in BB medium for 36 h, in the presence (red) or absence (gray) of the formate gradient, as determined using the plate-counting method. (C) Bacterial numbers of WT and ΔTlp1 cells cocultured for 36 h, in the presence (red) or absence (gray) of the formate gradient. The two strains were initially inoculated at a ratio of 1:1. (D) The ratio of WT and ΔTlp1 cells in the presence (orange) or absence (gray) of the formate gradient, as calculated based on the bacterial numbers in the coculture shown in panel C. (E) Bacterial numbers of C. jejuni WT and ΔCheY cells grown individually in BB medium for 36 h, under unstirred conditions, in the presence (red) or absence (gray) of the formate gradient. (F) Bacterial numbers of WT and ΔCheY cells cocultured for 36 h, in the presence (red) or absence (gray) of the formate gradient. The two strains were initially inoculated at a ratio of 1:1. (G) The ratio of WT and ΔCheY cells in the presence (orange) or absence (gray) of the formate gradient, as calculated based on the bacterial numbers in the coculture shown in panel F. The *P* values were calculated using a paired *t* test. *, *P* < 0.05; **, *P* < 0.01; ***, *P* < 0.001. The error bars indicate the standard errors of five replicates. WT, wild type.

To better understand the physiological relevance of the observed chemotaxis toward formate, we analyzed the effect of formate on the growth of C. jejuni cultures under microaerobic conditions. As in our experiments, C. jejuni NCTC 11168 cells had better motile ability when grown in liquid Brucella Broth (BB) medium, compared to Mueller-Hinton (MH) medium. We first measured the growth curves of C. jejuni at different concentrations of formate added to the BB medium under shaking conditions, and the results showed that formate promoted C. jejuni growth slightly at a concentration of 1 mM (Fig. S7A). Considering that the concentration of formate in BB medium is uniform under shaking conditions, we explored the potential fitness benefits of chemotaxis upon the introduction of a formate gradient into the unstirred culture using a small (40 μL) agarose bead that contained 30 mM formate. The experimental design is illustrated in Fig. 5A. Formate diffused from the agarose beads into the culture to form a concentration gradient under unstirred conditions. The growth of C. jejuni cells was determined by measuring the cell number in the stationary phase (at 36 h), which reflects cumulative differences in growth over the entire duration of culturing. The plate-counting method was used to measure the cell numbers in the culture. We discovered that upon culturing C. jejuni WT or ΔTlp1 individually, the growth of WT cells in the presence of the formate gradient was promoted significantly, compared to that observed in the control, which contained an agarose bead composed of only buffer, whereas the presence of the formate gradient did not affect the growth of the ΔTlp1 cells (Fig. 5B). Therefore, the Tlp1-mediated chemoattractant effect on the formate gradient may promote the growth of C. jejuni cells.

10.1128/mbio.03564-22.7 FIG S7 Growth of C. jejuni WT cells in different media in the presence of formate. (A) The growth of C. jejuni was measured with 1 mM formate added into the BB medium or without formate under shaking conditions. Samples were taken at the exponential and stationary stages of growth at 24 h, 36 h, and 48 h to detect the OD_600_ values. Error bars represent the standard errors of three replicates. (B) The growth of C. jejuni was measured with 1 mM formate added into the MH medium or without formate under shaking conditions. Samples were taken at the exponential and stationary stages of growth at 9 h, 21 h, and 33 h to detect the OD_600_ values. Error bars represent the standard errors of three replicates. Download FIG S7, TIF file, 0.5 MB. Copyright © 2023 Duan et al. 2023 Duan et al. https://creativecommons.org/licenses/by/4.0/ This content is distributed under the terms of the Creative Commons Attribution 4.0 International license.

Next, we explored the potential advantages of chemotaxis by carrying out growth competition between C. jejuni WT and ΔTlp1 cells in unstirred cocultures. The WT and ΔTlp1 cells were initially inoculated at a ratio of 1:1 and cocultured in BB medium with or without a formate gradient (Fig. 5A). To monitor the ratio of the two strains in the cocultures, the cell numbers of the competing strains in the stationary phase of growth were determined via the plate-counting method, making use of the kanamycin resistance of ΔTlp1 to distinguish between them. The WT and ΔTlp1 strains had similar cell densities in the stationary phase when inoculated with each strain alone, without formate (Fig. 5B). However, when the formate gradient was introduced into the WT and ΔTlp1 cocultures using the formate-containing agarose beads described above, the WT strain consistently outgrew the ΔTlp1 cells in the coculture (Fig. 5C), and the ratio of WT to ΔTlp1 cell numbers increased from 1.0 to 2.3 (Fig. 5D). In the absence of a formate gradient, the fractions of the two strains were similar (Fig. 5C), and the ratio of the cell numbers was maintained around 1.0 (Fig. 5D), indicating that the large growth advantage of the WT, relative to the ΔTlp1 cells, in the cocultures relies on the formate gradient. As a negative control, a propionate gradient was introduced into the culture via a similar method. The C. jejuni cells did not exhibit chemotaxis to propionate at the tested concentrations (Fig. S5C), and it is possible that they cannot utilize propionate (40). We found that the propionate gradient had no effect on the growth of either WT or ΔTlp1 cells when cultured with each strain individually (Fig. S8A) or with fractions of the WT and ΔTlp1 strains in the coculture (Fig. S8B and C).

10.1128/mbio.03564-22.8 FIG S8 The role of propionate and chemotaxis to formate in the growth of C. jejuni. (A) Bacterial numbers, as determined via the plate counting method, of C. jejuni WT and ΔTlp1 growing alone in the BB medium for 36 h in the presence (blue) or absence (grey) of a propionate gradient. (B) Bacterial numbers of WT and ΔTlp1 coculturing for 36 h in the presence (blue) or absence (grey) of a propionate gradient. The two strains were initially inoculated at a 1:1 ratio. (C) The ratio, calculated based on the bacterial numbers in the coculture shown in panel B, of WT and ΔTlp1 cells in the presence (green) or absence (grey) of a propionate gradient. In panels A to C, the source concentration of propionate in the bead was 30 mM. The *P* values were calculated using paired *t*-tests. The “ns” means no significance, compared to the buffer. Error bars indicate the standard errors of five replicates. (D) Bacterial numbers, as determined via the plate counting method, of C. jejuni WT and ΔTlp1 growing alone in the MH medium for 36 h in the presence (red) or absence (grey) of a formate gradient. (E) Bacterial numbers of WT and ΔTlp1 coculturing for 36 h in the presence (red) or absence (grey) of a formate gradient in the MH medium. The two strains were initially inoculated at a 1:1 ratio. (F) The ratio, calculated based on the bacterial numbers in the coculture shown in panel E, of WT and ΔTlp1 cells in the presence (orange) or absence (grey) of a formate gradient. In panels D to F, the source concentration of formate in the bead was 30 mM. The *P* values were calculated using paired *t*-tests. *, *P* < 0.05; **, *P* < 0.01. Error bars indicate the standard errors of four replicates. Download FIG S8, TIF file, 0.7 MB. Copyright © 2023 Duan et al. 2023 Duan et al. https://creativecommons.org/licenses/by/4.0/ This content is distributed under the terms of the Creative Commons Attribution 4.0 International license.

To further confirm that the growth advantage of C. jejuni WT cells in the presence of a formate gradient is due to chemotaxis, we performed similar experiments using the WT strain and the motile but nonchemotactic ΔCheY strain. The ΔCheY cells lost their chemotactic response to formate (Fig. S5B). When WT and ΔCheY cells were cultured individually with a formate gradient having been introduced into the culture, the growth of WT cells was promoted, as in previous experiments (Fig. 5B), whereas the growth of ΔCheY cells was not different due to the loss of chemotactic responses to formate (Fig. 5E). In the coculture of WT and ΔCheY cells, the increase in the ratio of WT to ΔCheY cells was approximately twofold after the introduction of the formate gradient (Fig. 5F and G). These results indicated that the growth advantage of the WT cells in the presence of a formate gradient, relative to the ΔTlp1 and ΔCheY cells, is dependent on the Tlp1-mediated chemoattractant responses toward formate.

We also conducted the growth experiments in MH medium, in which the nutrient is limited, compared to BB medium. Similar to the cells grown in BB medium, formate slightly promoted C. jejuni growth in MH medium supplemented with formate under shaking conditions (Fig. S7B). However, when culturing C. jejuni WT in unstirred MH medium, the growth of WT cells in the presence of the formate gradient was largely promoted (Fig. S8D). In the coculture of WT and ΔTlp1 cells, the increase in the ratio of WT to ΔTlp1 cells was approximately threefold after the introduction of the formate gradient (Fig. S8E and F), indicating that the growth advantage of the WT cells, relative to the ΔTlp1 cells, in the presence of a formate gradient is also significant in MH medium. Thus, we provide direct evidence that the promotion of the growth of C. jejuni by formate is correlated with Tlp1-mediated chemotaxis toward formate.

## DISCUSSION

Formate is produced by the anaerobic fermentation by microbial communities in the animal gut (39, 41). It is a primary energy source and acts as an electron donor for C. jejuni, which might affect energy metabolism and might play a role in the optimization of the microaerobic survival of C. jejuni in the gastrointestinal tract of the host (36, 38). Previous studies have shown that C. jejuni exhibits enhanced chemoattractive responses to formate, compared to other organic acids (29, 38). However, the chemoreceptors responsible for formate-sensing are still poorly understood. It has been reported that the two adjacent genes *cj0952c* and *cj0951c* might affect the chemotaxis of the C. jejuni isolate B2 toward formate (36). However, there is no evidence that the proteins encoded by these two genes are the chemoreceptors sensing formate. In this study, we found that formate is a ligand molecule for Tlp1, which is a conserved chemoreceptor in C. jejuni strains. Chemotaxis toward formate was observed in the C. jejuni WT strain NCTC 11168, but it was completely lost in the ΔTlp1 strain, suggesting that Tlp1 plays a predominant role in chemotaxis toward formate, compared to the *cj0952c*-encoded and *cj0951c*-encoded proteins.

Our study is the first to reveal the molecular mechanisms by which C. jejuni mediates its chemotaxis toward formate. The molecular docking prediction showed that formate binds directly to the membrane-proximal pocket of Tlp1-LBD, with its carboxyl group interacting with residues H251, Y287, and S290 through hydrogen bond formation. The selectivity of the membrane-proximal pocket in the dCache domain of Tlp1-LBD to recognize and trigger chemotaxis toward formate is high, as other organic acids or analogues, including acetate, propionate, and formaldehyde, bind weakly (*K_d_ *= 3.4 mM for acetate) (18) or show no binding to Tlp1-LBD, and therefore cannot elicit the chemotaxis of C. jejuni. A previous study showed that the respiratory inhibitors 2-*n*-heptyl-4-hydroxyquinoline *N*-oxide and sodium azide could not inhibit the attractive responses of C. jejuni toward formate (29), indicating that chemotaxis toward formate is not driven by metabolism-dependent energy taxis through oxidative phosphorylation but is instead driven by the direct sensing of formate by Tlp1-LBD, as shown in this study.

Although a previous report has suggested that formate could increase the growth of C. jejuni under microaerobic conditions (38), whether the promotion of growth is correlated with chemotaxis toward formate remains unknown. In the present study, we provide direct evidence of the physiological role of Tlp1-mediated chemotaxis toward formate in the promotion of C. jejuni growth. We found that a significant growth advantage conferred by formate chemotaxis could be observed, especially when C. jejuni WT and ΔTlp1 cells were competitively and statically cocultured with a formate gradient. Previous studies have reported that chemotaxis is important for the pathogenesis and colonization of the host for some enteric pathogens (10, 42–44), and the ability to metabolize specific nutrients enhances the pathogen colonization of specific tissues (45). The strong chemotaxis and utilization abilities of formate likely confer advantages to the gut colonization of C. jejuni. A reasonable deduction is that C. jejuni might orient itself and colonize regions with appropriate concentrations of formate in the gastrointestinal tract.

To our knowledge, C. jejuni Tlp1 is the first discovered chemoreceptor with a dCache domain that directly binds to formate with high specificity and elicits the strong chemotaxis of bacteria. The sCache chemoreceptor Atu0526 from A. fabrum C58 was reported to bind formate directly (32), but the residues in the binding pocket interacting with formate are different from those in the pocket of the dCache domain of C. jejuni Tlp1. A novel aspect of this study is that formate binds to the membrane-proximal subdomain of dCache. So far, Helicobacter pylori TlpC is the only reported chemoreceptor that binds to signals in the membrane-proximal subdomain (20). A few studies suggested that both dCache subdomains can bind ligands (22, 23). It is possible that the Tlp1 membrane-distal subdomain could bind other signals. A previous study indicated that the membrane-distal and membrane-proximal subdomains are closely related to each other and are structurally and dynamically coupled (46). It is interesting to explore how these two subdomains work together to sense different signals.

Our competitive experiments indicate that the chemotaxis to aspartate and formate via Tlp1 might be competitive. The metabolic pathway of aspartate in C. jejuni does not produce formate (47). Therefore, the physiological link between aspartate and formate in C. jejuni is not clear. As in our experiments, the E. coli cells expressing the hybrid receptor Tlp344Tar207 did not show a clear response to aspartate. Combining our results with the results of a prior report that showed that Tlp-LBD could not bind aspartate, as measured by ITC (18), we propose that Tlp1 senses aspartate through an indirect sensing mechanism, possibly through a periplasmic binding protein, as was suggested by the previous study. It is possible that Tlp1 integrates diverse signals in the single receptor through different sensing mechanisms to orient cells to find optimum niches.

The discovery of signaling molecules of C. jejuni chemoreceptors is of great importance in the study of host-pathogen interactions, pathogenesis, and colonization (48, 49). Although the ligand specificities of several C. jejuni chemoreceptors have been reported (17, 50), studies on the ligands of chemoreceptors and their roles in C. jejuni survival and growth are still rare. To our knowledge, 13 chemoreceptors have been reported in C. jejuni (9, 51). Therefore, it is difficult to screen the ligand molecules of individual chemoreceptors. In this study, we screened and successfully discovered a ligand of C. jejuni Tlp1 by constructing a functional hybrid receptor of it with E. coli Tar. Our results demonstrated that the ligands of individual target chemoreceptors can be efficiently discovered using hybrid receptors. Such a strategy for producing hybrid receptors could provide a universal methodology for the discovery of specific signaling molecules for other bacterial chemoreceptors.

## MATERIALS AND METHODS

### Bacterial strains, plasmids, and growth conditions.

The strains and plasmids used in the present study are listed in Table S2. C. jejuni strains were grown in either BB medium (2.2% brain heart infusion broth, 1% tryptone, and 0.2% yeast extract, pH 7.2; ExCell Bio, Shanghai, China) containing 10% fetal bovine serum or MH medium (0.6% beef powder, 0.15% soluble starch, and 1.75% acid hydrolyzed casein, pH 7.2; Hope Bio, Qingdao, China) for the chemotaxis and growth experiments, at 37°C, under microaerobic conditions (85% N_2_, 10% CO_2_, and 5% O_2_) (52). The E. coli strains were grown in either Luria-Bertani (LB) medium (Oxoid, USA) at 37°C for routine culture or tryptone broth (1% tryptone and 0.5% NaCl) at 34°C, for the chemotaxis experiments, under aerobic conditions. If required, the growth medium was supplemented with the appropriate antibiotics, including chloramphenicol (10 μg/mL for both C. jejuni and E. coli), kanamycin (10 μg/mL for both C. jejuni and E. coli), ampicillin (50 μg/mL for E. coli), and inducers (Table S2).

10.1128/mbio.03564-22.10 TABLE S2 Plasmids and bacterial strains used in this study. Download Table S2, DOCX file, 0.03 MB. Copyright © 2023 Duan et al. 2023 Duan et al. https://creativecommons.org/licenses/by/4.0/ This content is distributed under the terms of the Creative Commons Attribution 4.0 International license.

The plasmids pDJJ1, pDJJ2, and pDJJ3 were constructed to express the hybrid receptors. The coding sequence of each hybrid was amplified via PCR. The pKG116 plasmid was digested with NdeI and BamHI and was ligated with the amplified fragment, using the red/ET recombination of E. coli GB05-dir (53). The DNA fragment encoding the Tlp1-LBD (residues 31 to 326) was amplified via PCR and was ligated into the BamHI and NdeI digested plasmid pET28b to generate pET28b-Tlp1-LBD. The sequences of the Tlp1-LBD mutant proteins H251A, Y287A, and S290A were produced by introducing point mutations through primers and were recombined into DNA fragments via overlap PCR. They were then connected to the BamHI and NdeI digested plasmid pET28b. The plasmids pDJJ4, pDJJ5, and pDJJ6 were used to express the mutant hybrid receptors. Point mutations were introduced into the sequence using primers and were cloned into the NdeI and BamHI digested pKG116. All plasmids were verified via sequencing.

### Construction of the C. jejuni *tlp1-* and *cheY*-knockout strains.

The C. jejuni strain NCTC 11168 was used to generate the *tlp1*- and *cheY*- knockout strains. The knockout of the target gene was performed via double-crossover homologous recombination, using a suicide plasmid containing homology arms that flanked the target gene, as previously described (54). pBJ113, which was used as the initial knockout vector, was electrically transformed into E. coli GB08-red competent cells, and pBJ114 was produced via GB08-red mediated linear-circular homologous recombination (55) to replace the kanamycin resistance gene and the *galk* of pBJ113 with the chloramphenicol resistance gene and *sacB*, which were amplified from pBJ113 and pK18mobsacB, respectively. The linear *tlp1up-km*-*tlp1down* fragment, which contained 708 bp of the upstream *tlp1* gene, the kanamycin resistance gene, and 717 bp of the downstream *tlp1* gene, was connected via overlap PCR. The linear *cheYup-km*-*cheYdown* fragment contained 1 kb homologous arms of the upstream and downstream *cheY* genes and the kanamycin resistance gene. The fragment *tlp1up-km*-*tlp1down* or *cheYup-km*-*cheYdown* was transferred into E. coli GB08-red containing pBJ114 to produce pBJ115 or pBJ116. The inserted sequences in the transformants were verified via PCR screening and sequencing.

The resulting knockout vectors pBJ115 and pBJ116 were electroporated into C. jejuni using a modified protocol that was adapted from a previous report (56). Briefly, C. jejuni WT cells were first inoculated onto an MH agar plate and grown for 24 h, at 37°C, under microaerophilic conditions (5% O_2_, 10% CO_2_, and 85% N_2_). The harvested cells were pipetted onto fresh MH agar plates and grown for 18 h. The cells were then collected in MH broth, washed three times with washing buffer (15% [vol/vol] glycerol and 9% [wt/vol] sucrose) at 4°C, and resuspended to a final OD_600_ of 0.5. Plasmid DNA (2 μg) was added to 50 μL of C. jejuni competent cells and electroporated at 1.8 kV, 250 Ω, and 25 μF in an electrotransformer (Bio-Rad, USA) for approximately 5 ms. The cell suspension in super optimal broth with catabolite repression (SOC) medium was pipetted onto MH agar plates without any antibiotics. After overnight incubation, the cells were harvested and plated onto fresh MH agar that was supplemented with 10 μg/mL kanamycin. After 2 days of incubation, the cells were transferred to MH plates containing 10% sucrose to facilitate the double exchange of homologous recombination and completely remove the knockout plasmid. Single colonies of C. jejuni Δ*tlp1*::*km* and Δ*cheY*::*km* were verified via PCR and DNA sequencing.

### Complementation of the Δ*tlp1* mutant strain with the *tlp1* gene.

The insertion site for *tlp1* was selected between the 16S RNA and 23S RNA in the Δ*tlp1* mutant strain. Full-length *tlp1*, with the promoter region as well as the upstream 1,044 bp and the downstream 1,026 bp of the insertion site, was amplified from the genome. The linear fragment *16sRNAup*-*tlp1*-*cm*-*23sRNAdown* was generated via overlap PCR and electroporated into E. coli GB05-dir together with the amplified linear vector pBJ113, which contained only *sacB* and the origin of replication. Linear plus linear homologous recombination was performed using red/ET (53) to generate the plasmid pBJ110. Further screening was performed on LB plates containing chloramphenicol, and the plasmid was confirmed using DNA sequencing. Finally, pBJ110 was electroporated into C. jejuni Δ*tlp1*::*km* competent cells. *tlp1* complement cells were screened on chloramphenicol plates and verified via sequencing.

### Microfluidic experiments.

The E. coli cells expressing the hybrid receptor and enhanced green fluorescent protein (eGFP) protein were incubated at 34°C, with shaking (250 rpm), to an OD_600_ of 0.6. The cells were harvested via centrifugation at 3,000 rpm for 5 min, washed twice with tethering buffer (10 mM KH_2_PO_4_, 10 mM K_2_HPO_4_, and 0.1 mM EDTA, pH 7.0), and then resuspended in tethering buffer to an OD_600_ of 4.0. The compounds in the Biolog plates were dissolved in 50 μL water to a final concentration of 10 to 20 mM. The other compounds were dissolved in tethering buffer and adjusted to a pH of 7.0. The chemotactic responses of E. coli cells to various compounds were measured using a microfluidic device, as described previously (13, 57). After adding 4% agarose to fill the agarose channels, the collected E. coli cells were added to the sink pore of the device and freely diffused into the observation channel for 40 min. The compound solution was then added to the source pore and allowed to diffuse into the observation channel to establish a concentration gradient. The fluorescence intensity in the observation channel was detected using a 20× objective lens on a LSM 800 laser scanning confocal microscope (Zeiss, Germany). The CI was characterized by the fluorescence intensity in the analysis region of the observation channel, in response to the tested compound, normalized to that of the buffer channel. The data were analyzed using the ImageJ software package (Wayne Rasband, National Institutes of Health, USA). To measure the responses of the E. coli cells expressing the mutant hybrid receptor to formate, cells were resuspended in tethering buffer to an OD_600_ of 5.5 and added to the sink pore. Then, different concentrations of formate were added to the source pore, and the same procedures of measurements and data analysis that are described above were performed.

The microfluidic device described above was used to quantify the chemotaxis of the C. jejuni strain NCTC 11168. We used the BB medium for the chemotaxis experiments, as in our experiments, C. jejuni NCTC 11168 cells had better motility when grown in this medium. C. jejuni cells were cultured in BB medium, at 37°C, with shaking (100 rpm), under microaerobic conditions, to an OD_600_ of 0.2. The cells were harvested via centrifugation (3,000 rpm for 5 min), resuspended in phosphate-buffered saline (137 mM NaCl, 2.7 mM KCl, 4.3 mM Na_2_HPO_4_, and 1.5 mM KH_2_PO_4_, pH 7.2), to an OD_600_ of 2.0, and added in the sink pore of the microfluidic device to allow them to diffuse into the observation channel. The compound solution was then added to the source pores to form a concentration gradient. The chemotaxis of C. jejuni to the compound gradient was visualized using a 40× objective lens in the phase-contrast mode of an inverted fluorescence microscope (TI-E, Nikon, Japan). The experiments of aspartate competition were performed by incubating C. jejuni cells with 10 mM aspartate and keeping 10 mM aspartate throughout the chemotaxis assay to measure the response to formate.

### Expression and purification of the Tlp1-LBD protein.

For the expression of the recombinant protein, E. coli BL21(DE3) cells were transformed with the plasmid pET28b-Tlp1-LBD or its mutant. An overnight culture of E. coli BL21(DE3) containing pET28b-Tlp1-LBD or its mutant was inoculated into 100 mL LB medium with 10 μg/mL kanamycin and grown at 37°C, with shaking at 200 rpm. Expression was induced using 500 μM IPTG when the OD_600_ reached 0.6 to 0.8, and the cells were then grown overnight at 18°C, with shaking at 110 rpm.

E. coli cells expressing the Tlp1-LBD protein were harvested at 6,000 rpm and 4°C for 10 min. The cell pellets were resuspended in 50 mL buffer A (25 mM Na_2_HPO_4_, 25 mM NaH_2_PO_4_, and 500 mM NaCl, pH 8.0) and were lysed using an ultrahigh-pressure homogenizer (JNBIO, Guangzhou, China). The crushed cells in solution were centrifuged at 20,000 rpm and 4°C for 1 h to remove the insoluble cell debris. The clarified supernatant was added to a 5 mL HisTrap column (GE Healthcare, USA) that had been preequilibrated with buffer A. The column was washed with different concentrations of buffer B (25 mM Na_2_HPO_4_, 25 mM NaH_2_PO_4_, 500 mM NaCl, and 500 mM imidazole, pH 8.0), and the eluted protein was verified using sodium dodecyl sulfate-polyacrylamide gel electrophoresis. The residual imidazole in the protein solution was removed using a desalting column (GE Healthcare) with desalting buffer (25 mM Na_2_HPO_4_, 25 mM NaH_2_PO_4_ and 150 mM NaCl, pH 8.0), and the target protein was concentrated using a 30 kDa centrifugal filter (Merck Millipore, USA). The Tlp1-LBD mutant proteins, namely, H251A and Y287A, were purified using methods similar to those described above. Because Tlp1-LBD S290A was insoluble, we used the SUMO fusion protein tag to promote its expression and purification.

### Circular dichroism spectroscopy.

The purified Tlp1-LBD and its mutants were buffer-exchanged into desalting buffer (25 mM Na_2_HPO_4_, 25 mM NaH_2_PO_4_, pH 8.0) without chloride ions by using an ultrafiltration tube. Far-UV circular dichroism (CD) spectra were recorded at a protein concentration of 0.048 mg/mL at 25°C, at a scanning speed of 100 nm/min, in the wavelength range of 190 nm to 260 nm, using a JASCO J-1500 circular dichroism spectrometer. The data were solvent corrected. The curves were smoothed using the Prism GraphPad 8.0.2 software package.

### Analyses of the ligand-binding pockets of Tlp1-LBD and the molecular docking with the ligand.

The Tlp1-LBD crystal structure was derived from the PDB database (4WY9). A site-specific binding pocket analysis was performed using the PyMOL software plugin PyVOL. The parameters were set to a maximum probe of 3.4 Å and a minimum probe of 0.5 Å. AutoDock software (58) was used to perform the molecular docking of Tlp1-LBD with formate. The membrane-distal and membrane-proximal pockets of Tlp1-LBD that are analyzed above were used as the docking sites for formate. The binding free energy of each binding conformation was calculated to obtain the minimum binding energy and the best configuration. The three-dimensional structures were displayed using PyMOL.

### ITC.

ITC experiments were performed at 25°C, using a PEAQ ITC isothermal titration calorimeter (Malvern Instruments, United Kingdom). The Tlp1-LBD protein, its mutants, and formate were dissolved in desalting buffer (25 mM Na_2_HPO_4_, 25 mM NaH_2_PO_4_, and 150 mM NaCl, pH 8.0). In each experiment, 350 μL of Tlp1-LBD protein, H251A, Y287A, or S290A were added to the sample pool and titrated with a formate solution at the indicated concentrations. Binding isotherms were generated by plotting the thermal change that was generated by each injection against the molar ratio of formate to Tlp1-LBD, H251A, Y287A, or S290A. As a control, formate was titrated into buffer without protein. The data were fitted to a one-site binding model of the MicroCal PEAQ ITC Analysis software (Malvern Instruments).

### The effect of chemotaxis on C. jejuni growth.

To investigate the effect of chemotaxis toward formate on the growth of C. jejuni under shaking conditions, the cells were inoculated into BB or MH medium with an initial OD_600_ of 0.1. The cells were cultured with or without 1 mM formate at 37°C and 100 rpm, under microaerobic conditions (5% O_2_, 10% CO_2_, and 85% N_2_). The OD_600_ values of the C. jejuni cultures were determined after inoculation.

To explore the fitness benefits of chemotaxis under unstirred conditions with the formate gradient, a small (40 μL) agarose bead that contained 30 mM formate was added to 3 mL of BB or MH medium to generate a formate gradient. The beads were immobilized below the liquid layer of the culture medium using a previously reported method (59). In the presence or absence of a formate gradient, 10 μL of C. jejuni cell solution (WT, ΔTlp1, or ΔCheY) was inoculated individually into 3 mL of medium. After growing for 36 h under static conditions, 100 μL of the dilution were spread onto MH agar plates or plates containing kanamycin (10 μg/mL) to determine the CFU of each strain. In the growth competition experiments, two competing C. jejuni strains (WT:ΔTlp1 and WT:ΔCheY) were inoculated at a ratio of 1:1 into 3 mL of medium, with or without a formate gradient. To monitor the ratio of the two competing strains in the cocultures under unstirred conditions, the cell numbers of the two strains were determined via the plate-counting method, after a 36 h culture, while distinguishing the C. jejuni knockout strain from the WT on the basis of its kanamycin resistance.

### Motility assays for the C. jejuni WT and mutant strains.

To determine the motility of the C. jejuni cells, WT and ΔTlp1 cells were first cultured on MH agar plates at 37°C, for 18 to 24 h, under microaerobic conditions. The cells were then collected and resuspended in MH medium until an OD_600_ of 0.05 was reached. The cell solutions (2 μL) were pipetted into semisolid MH agar (0.4% agar) plates and incubated at 37°C, for 48 h. Motility was assessed by measuring the diameters of the swimming zones.

### Data availability.

All data generated or analyzed during this study are included in this article and its supplemental information files.
