# Supplementary figures and images for: The dCache Domain of the Chemoreceptor Tlp1 in Campylobacter jejuni Binds and Triggers Chemotaxis toward Formate
Source: mBio. 2023 Apr 13;14(3):e03564-22. doi: 10.1128/mbio.03564-22 (PMC10294657; doi:10.1128/mbio.03564-22)

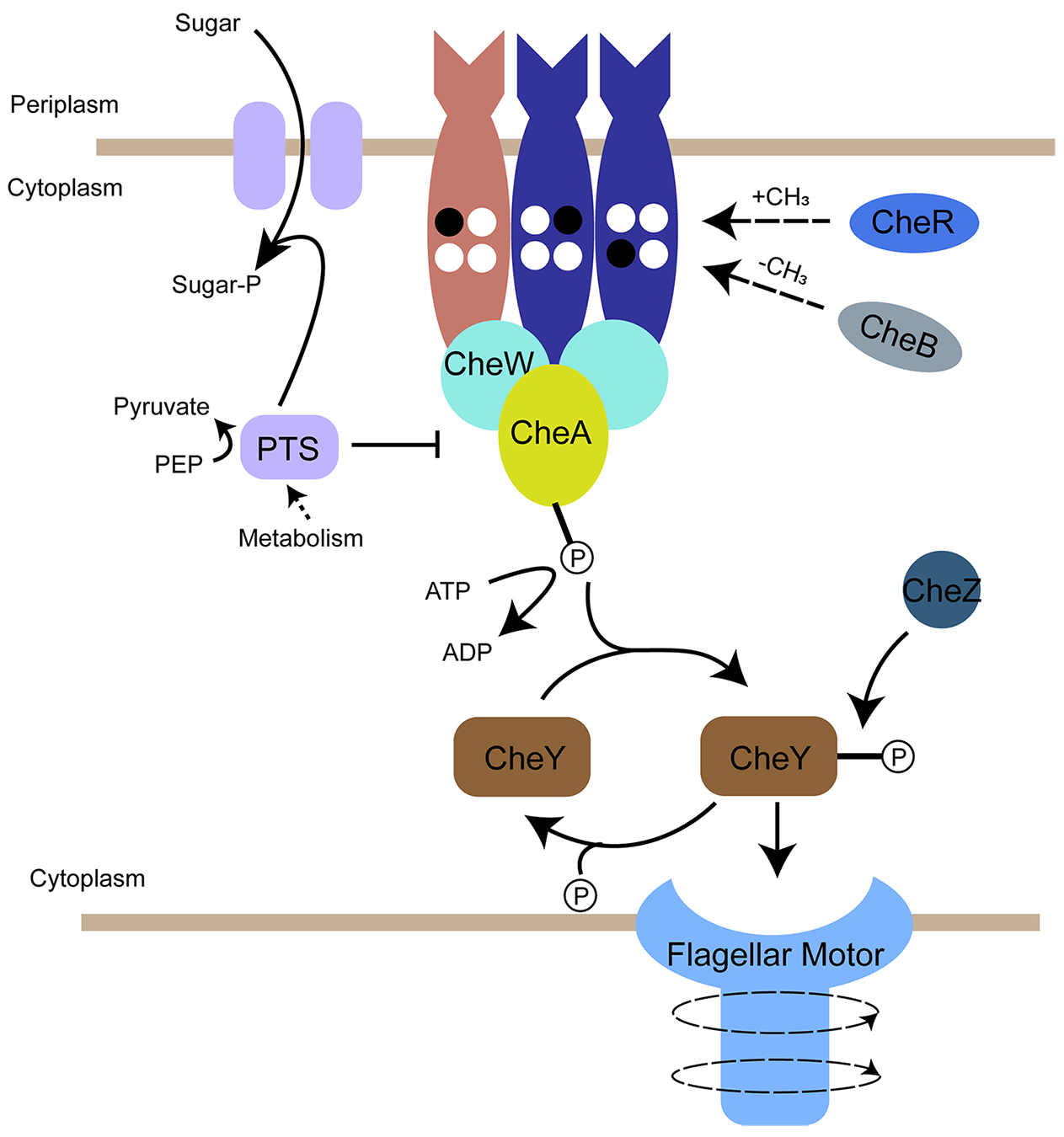

Supplement: FIG S1 [file mbio.03564-22-s0001.tif]

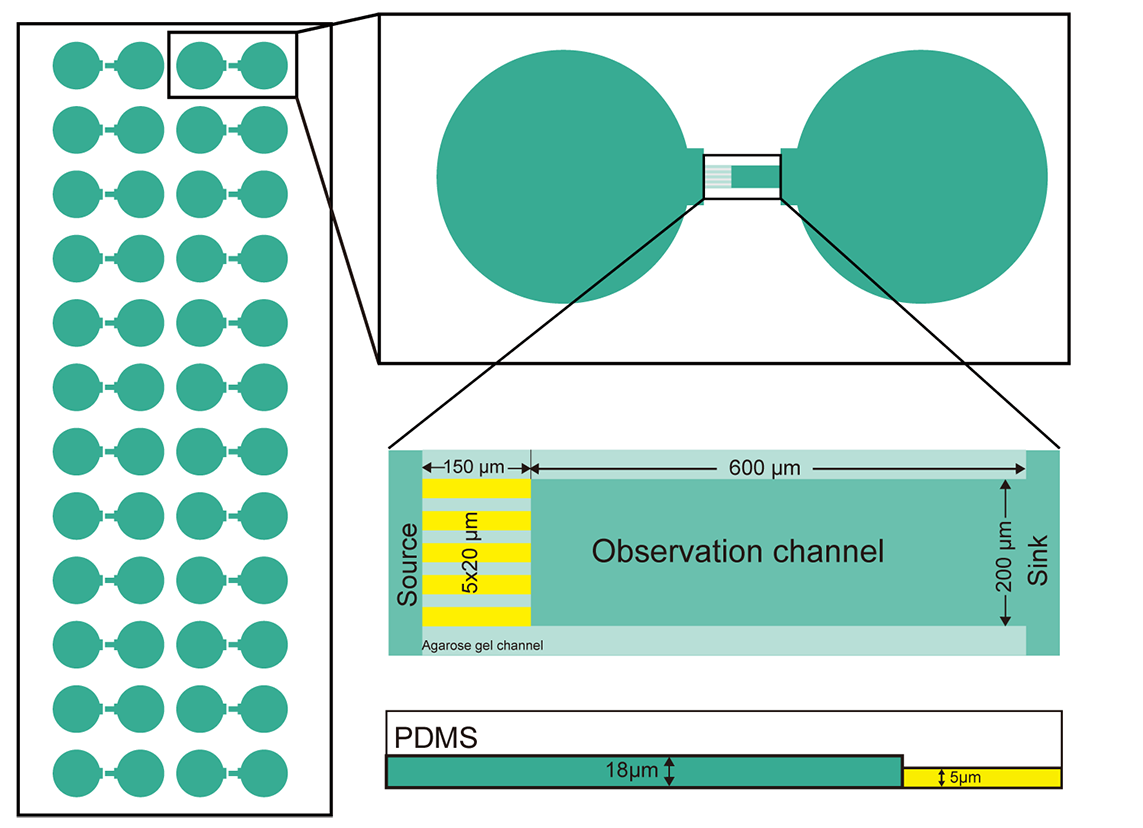

Supplement: FIG S2 [file mbio.03564-22-s0002.tif]

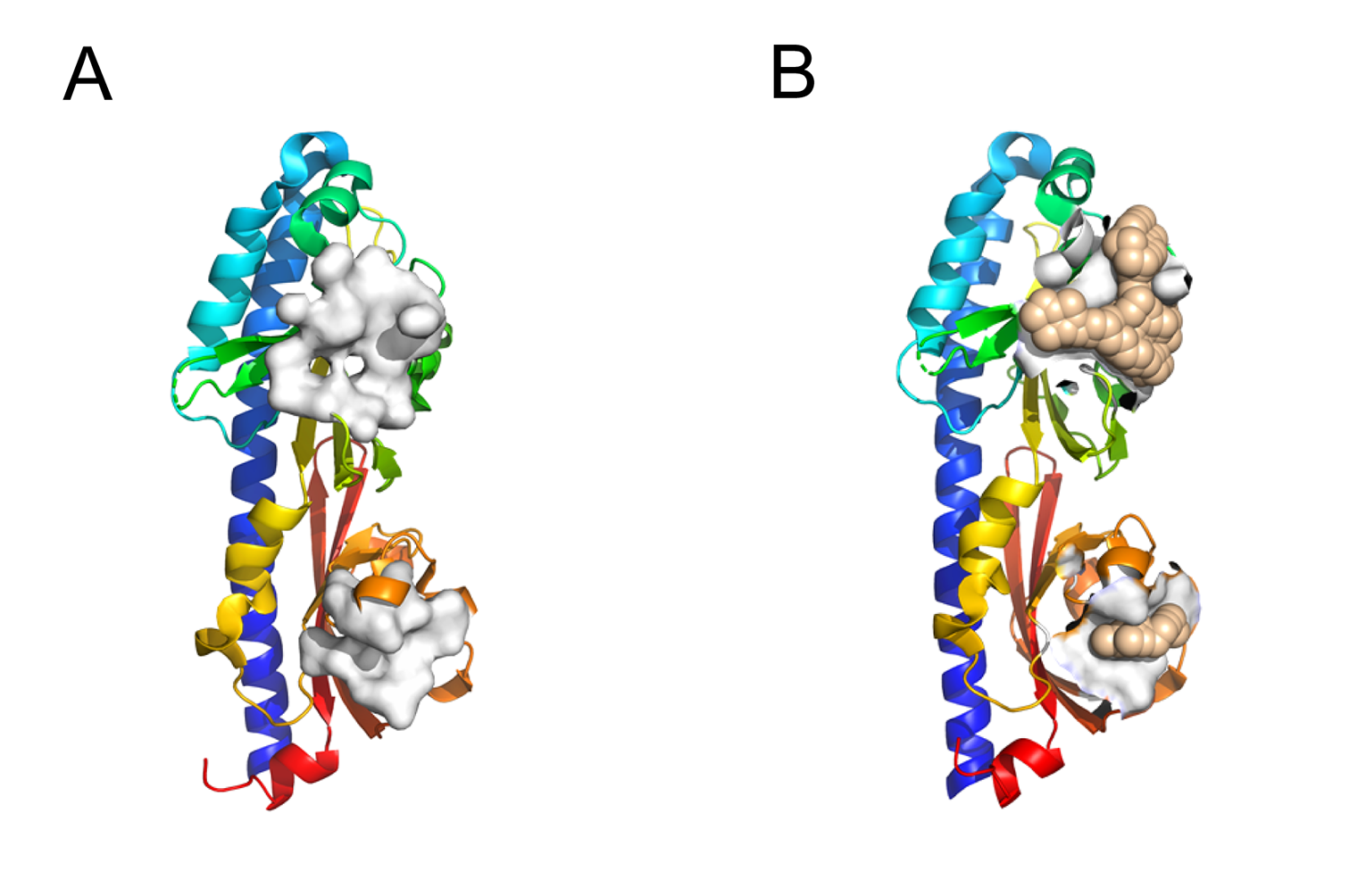

Supplement: FIG S3 [file mbio.03564-22-s0003.tif]

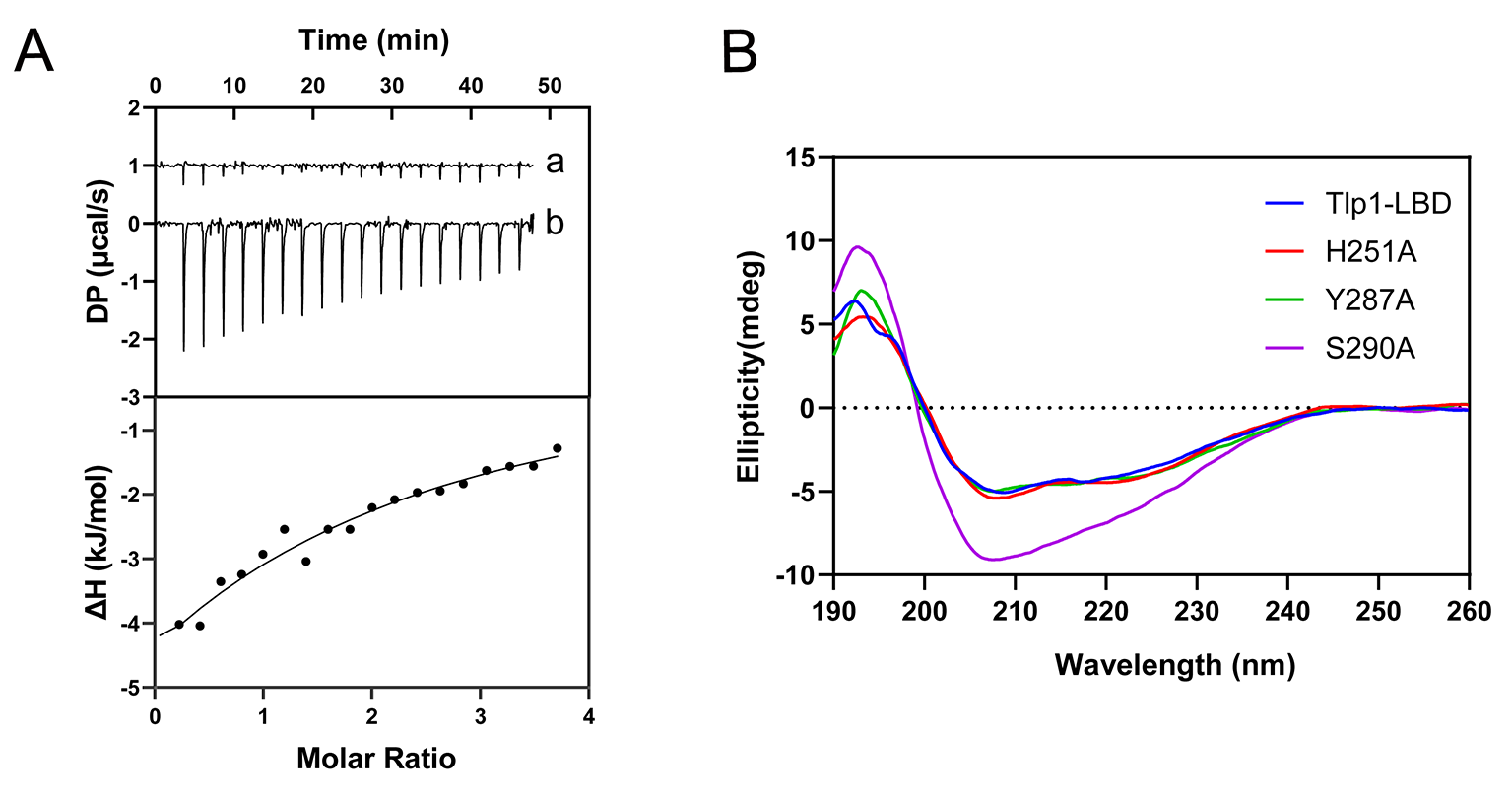

Supplement: FIG S4 [file mbio.03564-22-s0004.tif]

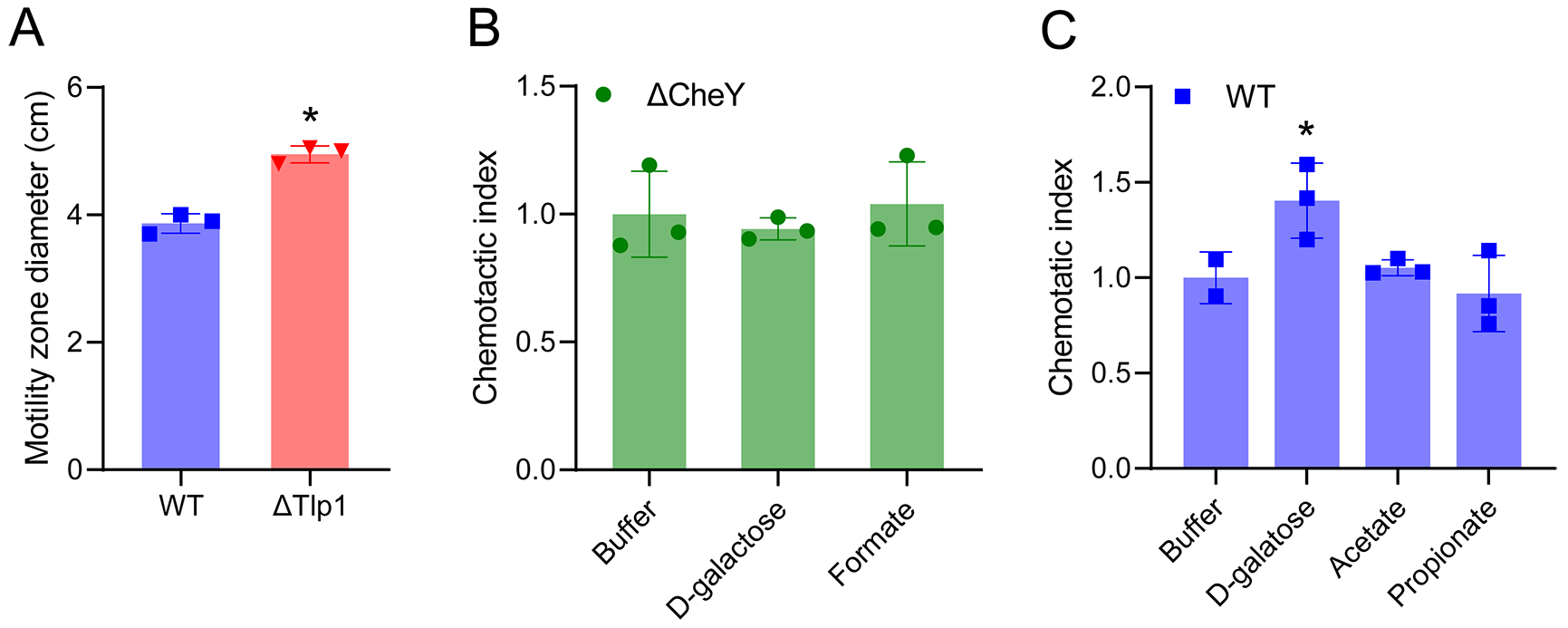

Supplement: FIG S5 [file mbio.03564-22-s0005.tif]

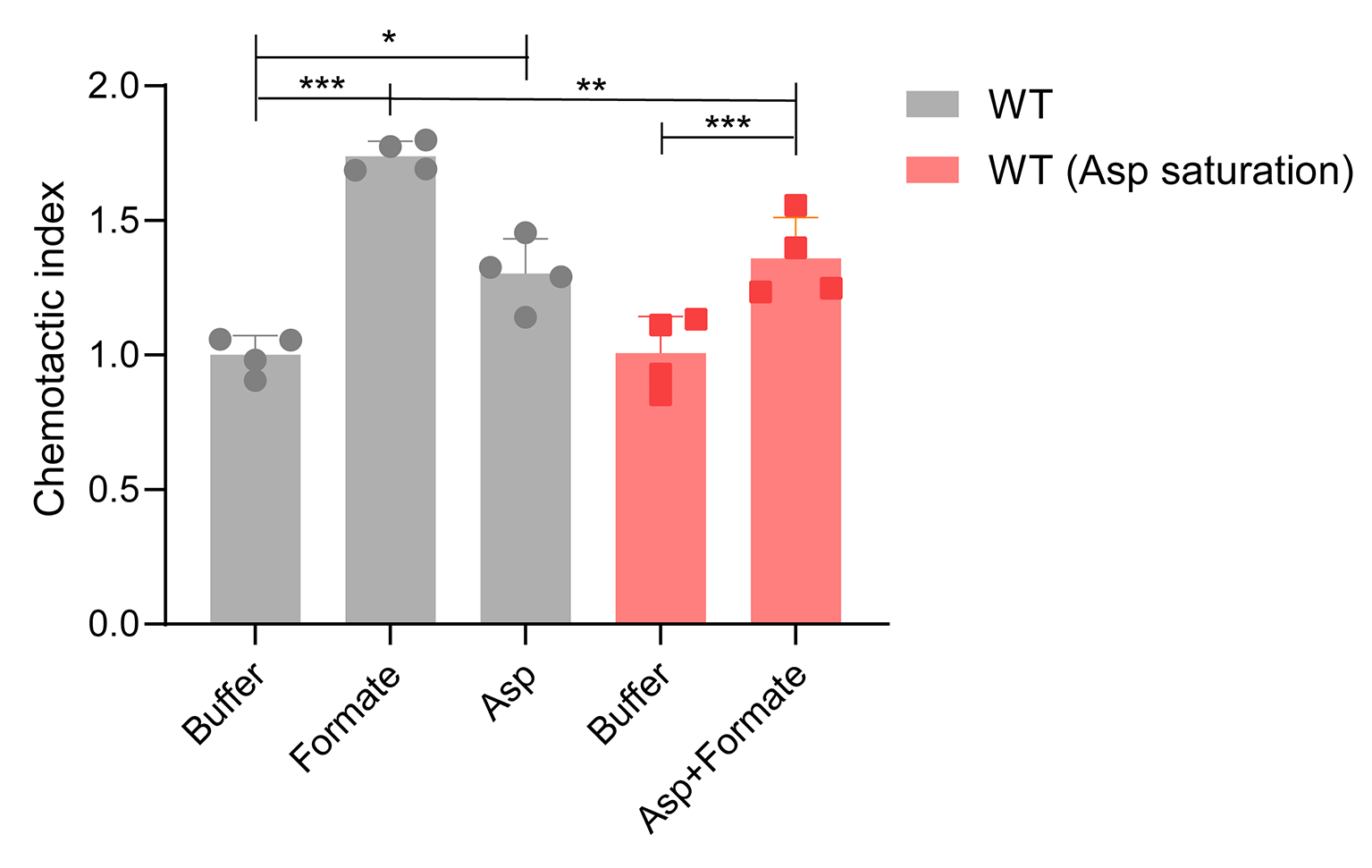

Supplement: FIG S6 [file mbio.03564-22-s0006.tif]

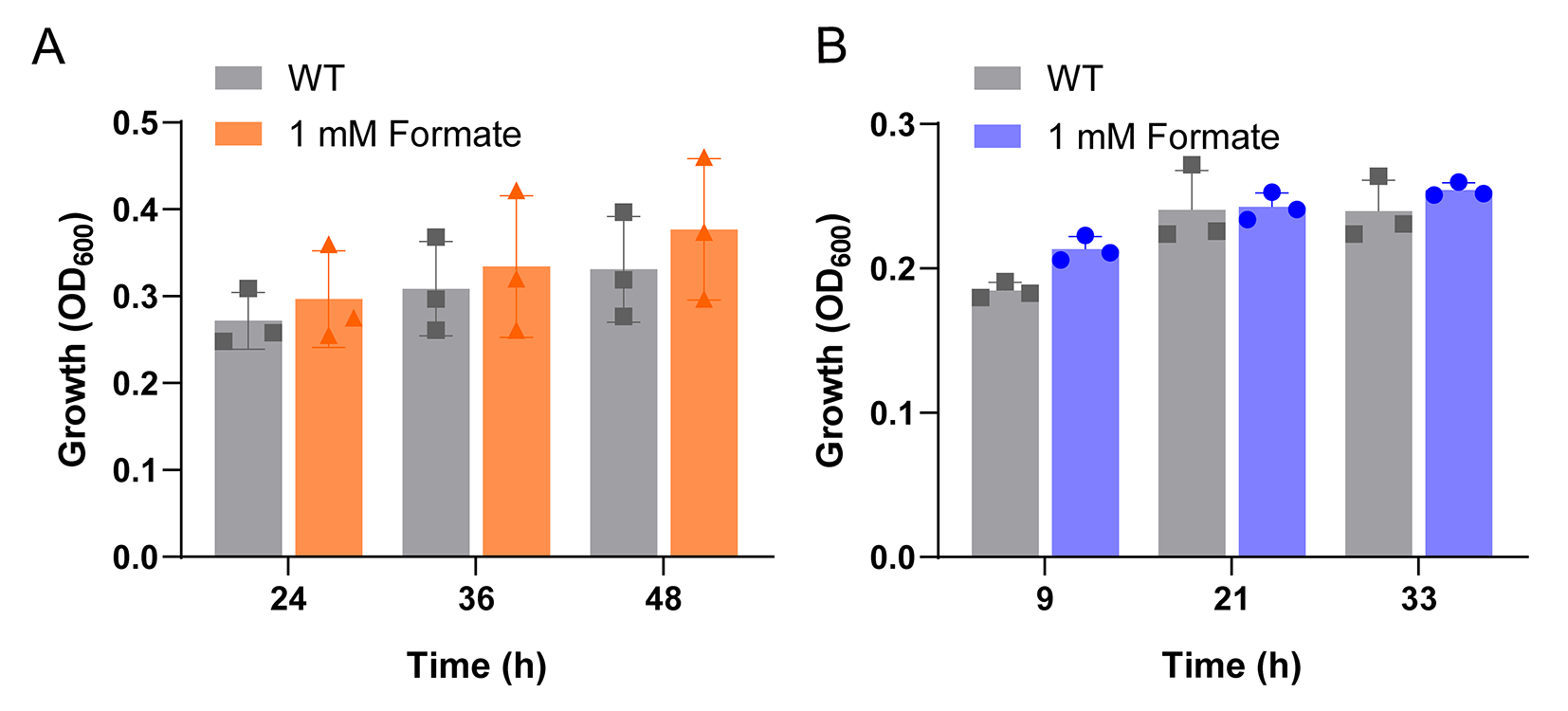

Supplement: FIG S7 [file mbio.03564-22-s0007.tif]

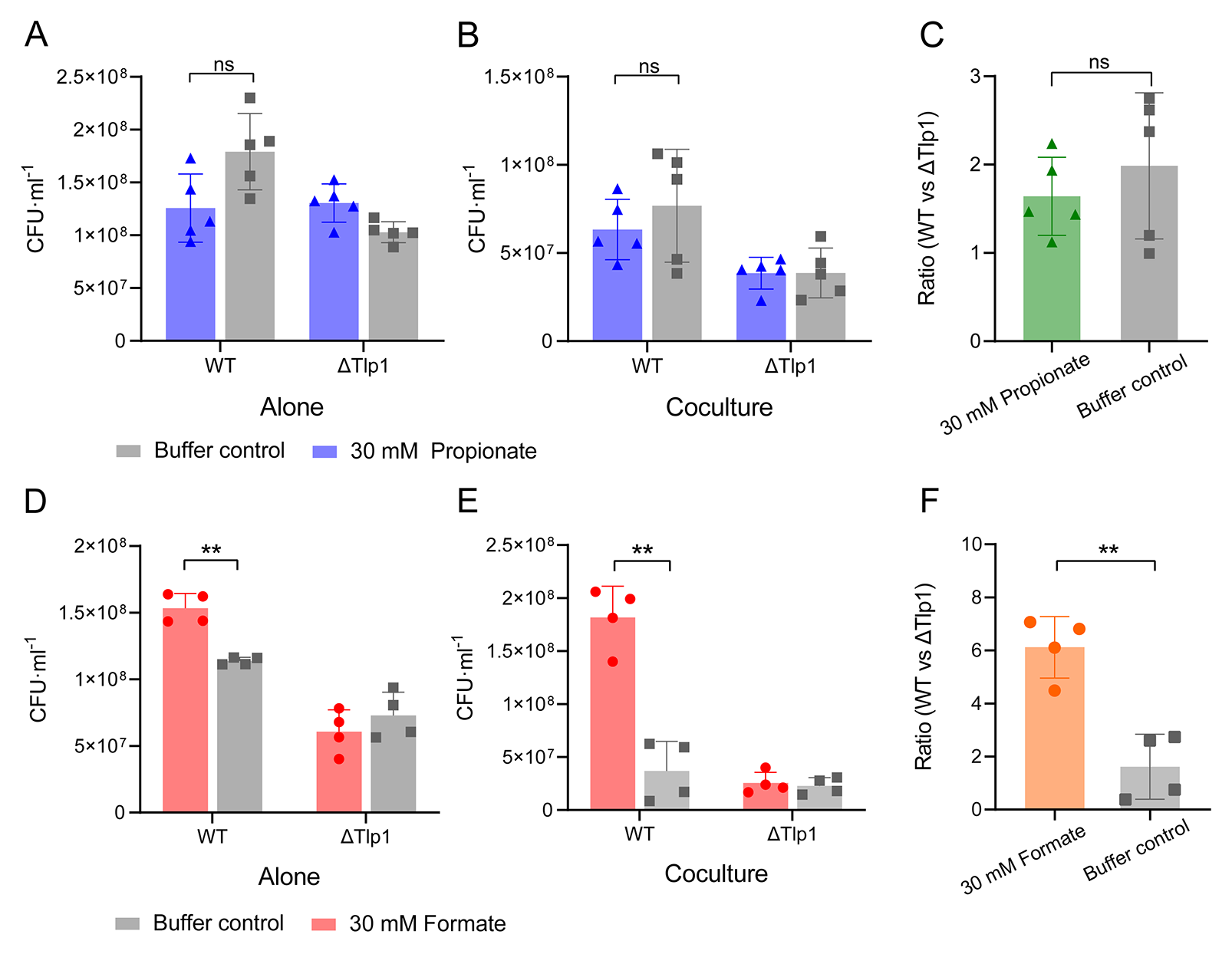

Supplement: FIG S8 [file mbio.03564-22-s0008.tif]
