# Supplementary material for: The dCache Domain of the Chemoreceptor Tlp1 in Campylobacter jejuni Binds and Triggers Chemotaxis toward Formate
Source: mBio. 2023 Apr 13;14(3):e03564-22. doi: 10.1128/mbio.03564-22 (PMC10294657; doi:10.1128/mbio.03564-22)
Supplement: TABLE S1 [file mbio.03564-22-s0009.docx]

**Table S1** Compounds used for ligand screening in this study

| **NO.** | **Name** | **Structure** | **NO.** | **Name** | **Structure** |
| --- | --- | --- | --- | --- | --- |
| 1 | L-Proline |  | 28 | Ethylenediamine |  |
| 2 | D-Alanine |  | 29 | Acetamide |  |
| 3 | Formate |  | 30 | 1,4-Butanediamine |  |
| 4 | α-Ketobutyric acid |  | 31 | *cis*-4-Hydroxy-L-proline |  |
| 5 | γ-Hydroxybutanoic acid |  | 32 | Nicotinic acid |  |
| 6 | Glycolic acid |  | 33 | Nicotinamide |  |
| 7 | Glyoxylic acid |  | 34 | Choline |  |
| 8 | Ethylamine |  | 35 | Valeric acid |  |
| 9 | Glycine |  | 36 | Pyruvic acid |  |
| 10 | L-Hydroxyproline |  | 37 | γ-Aminobutyric acid |  |
| 11 | Aspartic acid |  | 38 | Taurine |  |
| 12 | Leucine |  | 39 | Acetic acid |  |
| 13 | L-Pyroglutamic acid |  | 40 | Propanoic acid |  |
| 14 | Sec-butylamine |  | 41 | Butyric acid |  |
| 15 | 2,3-Butanediol |  | 42 | Isobutyric acid |  |
| 16 | Acetoin |  | 43 | Benzoic acid |  |
| 17 | Ammonia |  | 44 | D-Lactic acid |  |
| 18 | Urea |  | 45 | L-Lactic acid |  |
| 19 | Hydroxylamine |  | 46 | Malic acid |  |
| 20 | Methylamine |  | 47 | Succinic acid |  |
| 21 | Butylamine |  | 48 | L-Valine |  |
| 22 | 2-Aminoethanol |  | 49 | Ethanol |  |
| 23 | Histidine |  | 50 | IPA |  |
| 24 | β-Hydroxybutyric acid |  | 51 | L-Tartaric acid |  |
| 25 | 2,3-Butanedione |  | 52 | 3-Hydroxypropionic acid |  |
| 26 | D,L-Lactamide |  | 53 | Betaine |  |
| 27 | Formamide |  | 54 | Formaldehyde |  |
