# Supplementary material for: The dCache Domain of the Chemoreceptor Tlp1 in Campylobacter jejuni Binds and Triggers Chemotaxis toward Formate
Source: mBio. 2023 Apr 13;14(3):e03564-22. doi: 10.1128/mbio.03564-22 (PMC10294657; doi:10.1128/mbio.03564-22)
Supplement: TABLE S2 [file mbio.03564-22-s0010.docx]

**Table S2** Plasmids and bacterial strains used in this study

| **Strains or plasmids** | **Description** | **Antibiotic resistance** | **Induction** | **Reference or source** |
| --- | --- | --- | --- | --- |
| **Plasmids** |  |  |  |  |
| pKG116 | Protein expression plasmid | Cm | - | (1) |
| pTrc99a-EGFP | EGFP expression plasmid | Amp | 50 μM IPTG | (2) |
| pBJ113 | Gene replacement vector with Km and Galk cassette | Km | - | (3) |
| pET28b | Protein expression plasmid | Km | - | Novagen |
| pK18mobsacB | Suicide plasmid for deletion mutant construction | Km | - | (4) |
| pDJJ1 | Hybrid Tlp1[1-336]-Tar[200-553] expression plasmid, pKG116 derivative | Cm | 0.7 µM sodium salicylate | This study |
| pDJJ2 | Hybrid Tlp1[1-340]-Tar[203-553] expression plasmid, pKG116 derivative | Cm | 0.7 µM sodium salicylate | This study |
| pDJJ3 | Hybrid Tlp1[1-344]-Tar[207-553] expression plasmid, pKG116 derivative | Cm | 0.7 µM sodium salicylate | This study |
| pDJJ4 | Hybrid Tlp1[1-344]-Tar[207-553]-H251A expression plasmid, pKG116 derivative | Cm | 0.7 µM sodium salicylate | This study |
| pDJJ5 | Hybrid Tlp1[1-344]-Tar[207-553]-Y287A expression plasmid, pKG116 derivative | Cm | 0.7 µM sodium salicylate | This study |
| pDJJ6 | Hybrid Tlp1[1-344]-Tar[207-553]-S290A expression plasmid, pKG116 derivative | Cm | 0.7 µM sodium salicylate | This study |
| pET28b-Tlp1-LBD | Tlp1-LBD expression plasmid, pET-28b derivative | Km | 500 μM IPTG | This study |
| pET28b-Tlp1- LBD-H251A | Tlp1-LBD-H251A expression plasmid, pET-28b derivative | Km | 500 μM IPTG | This study |
| pET28b-Tlp1- LBD-Y287A | Tlp1-LBD-Y287A expression plasmid, pET-28b derivative | Km | 500 μM IPTG | This study |
| pET28b-Tlp1-LBD-S290A | Tlp1-LBD-S290A-SUMO expression plasmid, pET-28b derivative | Km | 500 μM IPTG | This study |
| pBJ114 | Gene replacement vector with Cm and SacB cassette, pBJ113 derivative | Cm | - | This study |
| pBJ115 | *tlp1* knockout plasmid, pBJ114 derivative | Km, Cm | - | This study |
| pBJ116 | *cheY* knockout plasmid, pBJ114 derivative | Km, Cm | - | This study |
| pBJ117 | *tlp1* complementing plasmid | Cm | - | This study |
| **Strains** |  |  |  |  |
| E. coli VS188 | Δ*aer*Δ*tsr*Δ*tar*Δ*tap*Δ*trg*, RP437 derivative | - | - | (5) |
| E. coli GB05-dir | (*fhu*A::IS2, Δ*ybcC*, Δ*recET*, P_BAD_-*ETγA*) with an arabinose-inducible *ETγA* operon (full-length *recE*, *recT*, *redγ* and *recA*), DH10B derivative | Sm | 17.5 mM  L-arabinose | (6) |
| E. coli GB08-red | (*fhu*A::IS2, Δ*ybcC*, Δ*recET*, P_RhaSR_-*γβαA*) with a rhamnose-inducible *γβαAA* operon (*redγ*, *redβ*, *redα* and *recA*), DH10B derivative | Sm | - | (6) |
| E. coli BL21(DE3) | F-ompT hsdSB(rB-mB-)gal dcm (DE3) | - | - | (7) |
| *C. jejuni* NCTC 11168 | *C. jejuni* wild-type strain | - | - | (8) |
| *C. jejuni* 11168/Δ*tlp1* | tlp1 knockout strain | Km | - | This study |
| *C. jejuni* 11168/Δ*tlp1c* | tlp1complemented strain in C. jejuni 11168/Δtlp1 | Cm | - | This study |
| *C. jejuni* 11168/Δ*cheY* | cheY knockout strain | Km | - | This study |

**References**

1. Burón-Barral MC, Gosink KK, Parkinson JS. 2006. Loss- and gain-of-function mutations in the F1-HAMP region of the *Escherichia coli* aerotaxis transducer Aer. J Bacteriol 188:3477-3486. <http://doi.org/10.1128/jb.188.10.3477-3486.2006>.

2. Bi S, Pollard AM, Yang Y, Jin F, Sourjik V. 2016. Engineering hybrid chemotaxis receptors in bacteria. ACS Synth Biol 5:989-1001. <http://doi.org/10.1021/acssynbio.6b00053>.

3. Zhuo L, Wan TY, Pan Z, Wang JN, Sheng DH, Li YZ. 2022. A dual-functional orphan response regulator negatively controls the differential transcription of duplicate groELs and plays a global regulatory role in *Myxococcus*. mSystems 7:e0105621. <http://doi.org/10.1128/msystems.01056-21>.

4. Wang P, Yu Z, Li B, Cai X, Zeng Z, Chen X, Wang X. 2015. Development of an efficient conjugation-based genetic manipulation system for *Pseudoalteromonas*. Microb Cell Fact 14:11. <http://doi.org/10.1186/s12934-015-0194-8>.

5. Ames P, Studdert CA, Reiser RH, Parkinson JS. 2002. Collaborative signaling by mixed chemoreceptor teams in *Escherichia coli*. Proc Natl Acad Sci U S A 99:7060-7065. <http://doi.org/10.1073/pnas.092071899>.

6. Fu J, Bian X, Hu S, Wang H, Huang F, Seibert PM, Plaza A, Xia L, Müller R, Stewart AF, Zhang Y. 2012. Full-length RecE enhances linear-linear homologous recombination and facilitates direct cloning for bioprospecting. Nat Biotechnol 30:440-446. <http://doi.org/10.1038/nbt.2183>.

7. Jeong H, Barbe V, Lee CH, Vallenet D, Yu DS, Choi SH, Couloux A, Lee SW, Yoon SH, Cattolico L, Hur CG, Park HS, Ségurens B, Kim SC, Oh TK, Lenski RE, Studier FW, Daegelen P, Kim JF. 2009. Genome sequences of *Escherichia coli* B strains REL606 and BL21(DE3). J Mol Biol 394:644-652. <http://doi.org/10.1016/j.jmb.2009.09.052>.

8. Marchant J, Wren B, Ketley J. 2002. Exploiting genome sequence: predictions for mechanisms of *Campylobacter* chemotaxis. Trends Microbiol 10:155-159. <http://doi.org/10.1016/s0966-842x(02)02323-5>.
